# Identification of biomolecule mass transport and binding rate parameters in living cells by inverse modeling

**DOI:** 10.1186/1742-4682-3-36

**Published:** 2006-10-11

**Authors:** Kouroush Sadegh Zadeh, Hubert J Montas, Adel Shirmohammadi

**Affiliations:** 1 Fischell Department of Bioengineering, University of Maryland, College Park, Maryland 20742, USA

## Abstract

**Background:**

Quantification of in-vivo biomolecule mass transport and reaction rate parameters from experimental data obtained by Fluorescence Recovery after Photobleaching (FRAP) is becoming more important.

**Methods and results:**

The Osborne-Moré extended version of the Levenberg-Marquardt optimization algorithm was coupled with the experimental data obtained by the Fluorescence Recovery after Photobleaching (FRAP) protocol, and the numerical solution of a set of two partial differential equations governing macromolecule mass transport and reaction in living cells, to inversely estimate optimized values of the molecular diffusion coefficient and binding rate parameters of GFP-tagged glucocorticoid receptor. The results indicate that the FRAP protocol provides enough information to estimate one parameter uniquely using a nonlinear optimization technique. Coupling FRAP experimental data with the inverse modeling strategy, one can also uniquely estimate the individual values of the binding rate coefficients if the molecular diffusion coefficient is known. One can also simultaneously estimate the dissociation rate parameter and molecular diffusion coefficient given the pseudo-association rate parameter is known. However, the protocol provides insufficient information for unique simultaneous estimation of three parameters (diffusion coefficient and binding rate parameters) owing to the high intercorrelation between the molecular diffusion coefficient and pseudo-association rate parameter. Attempts to estimate macromolecule mass transport and binding rate parameters simultaneously from FRAP data result in misleading conclusions regarding concentrations of free macromolecule and bound complex inside the cell, average binding time per vacant site, average time for diffusion of macromolecules from one site to the next, and slow or rapid mobility of biomolecules in cells.

**Conclusion:**

To obtain unique values for molecular diffusion coefficient and binding rate parameters from FRAP data, we propose conducting two FRAP experiments on the same class of macromolecule and cell. One experiment should be used to measure the molecular diffusion coefficient independently of binding in an effective diffusion regime and the other should be conducted in a reaction dominant or reaction-diffusion regime to quantify binding rate parameters. The method described in this paper is likely to be widely used to estimate in-vivo biomolecule mass transport and binding rate parameters.

## Background

Transport of biomolecules in small systems such as living cells is a function of diffusion, reactions, catalytic activities, and advection. Innovative experimental protocols and mathematical modeling of the dynamics of intracellular biomolecules are key tools for understanding biological processes and identifying their relative importance. One of the most widely used techniques for studying in vitro and in vivo diffusion and binding reactions, nuclear protein mobility, solute and biomolecule transport through cell membranes, lateral diffusion of lipids in cell membranes, and biomolecule diffusion within the cytoplasm and nucleus, is Fluorescence Recovery after Photobleaching (FRAP). The technique was developed in the 1970s and was initially used to study lateral diffusion of lipids through the cell membrane [1-9]. At the time, biophysicists paid little attention to the procedure, but since the invention of the Green Fluorescent Protein (GFP) technique, also known as GFP fusion protein technology, and the development of the commercially available confocal-microscope-based photobleaching methods, its applications have increased drastically [10-14]. A detailed description of the protocol is presented in [13,15].

The number and complexity of quantitative analyses of the FRAP protocol have increased over the years. Early analyses characterized diffusion alone [7,16-18]. More recently, investigators have studied the interaction of GFP-tagged proteins with binding sites inside living cells [11,19]. Some have considered faster and slower recovery as measures of weaker and tighter binding, respectively. By analyzing the shape of a single FRAP curve, others have tried to draw conclusions about the underlying biological processes [12,13,20]. Ignoring diffusion and presuming a full chemical reaction model, some researchers have performed quantitative analyses to identify pseudo-association and dissociation rate coefficients [16,18,20-24].

To describe diffusion-reaction processes in the FRAP protocol, one needs to solve the full diffusion-reaction model. Sprague et al. [14] presented an analytical treatment of the diffusion-reaction model and stated where pure diffusion, pure reaction, and diffusion-reaction regimes are dominant. They used the model to simulate the mobility of the GFP-tagged glucocorticoid receptor (GFP-GR) in nuclei of both normal and ATP-depleted cells. Using the mass of GFP-GR, they assumed a free molecular diffusion coefficient of 9.2 *μm*^2 ^*s*^-l ^for GFP-GR and fitted two binding rate parameters by curve fitting. On the basis of these parameters they concluded that GFP-GR diffuses from one binding site to the next with an average time of 2.5 ms; the average binding time per site is 12.7 ms. They also concluded that 14% of the GFP-GR is free and 86% is bound. There have been other theoretical investigations of full diffusion-reaction models in FRAP experiments [10,25,26].

What is missing from these comprehensive FRAP analyses is a robust and systematic method for extracting as much physiochemical information from the protocol as possible and for quantifying the related parameters. There are several in vivo and in vitro methods for measuring mass transport and reaction rate parameters. However, in vitro results may not be representative of in vivo transport processes. In-vivo measurements, on the other hand, often impose unrealistic and simplified initial and boundary conditions on transport processes in biological systems. Also, information regarding parameter uncertainty is not readily obtained from these methods unless a very large number of samples and measurements are taken at significant additional cost [27].

To overcome these limitations, indirect methods such as parameter optimization by inverse modeling can be used to identify mass transport and biochemical reaction rate parameters. Inverse modeling is usually defined as estimation of model parameters by matching a numerical or analytical model to observed data representing the system response at a discrete time and location. In other words, "inverse problems are those where a set of measured results is analyzed in order to get as much information as possible on a 'model' which is proposed to represent a system in the real world" [28]. Inverse techniques usually combine a numerical or analytical model with a parameter optimization algorithm and experimental data set to estimate the optimum values of model parameters, imposed initial and boundary condition and other properties of the excitation-response relationship of the system under study. The technique searches iteratively for the best combination of parameter values, by varying the unknown coefficients and comparing the measured response of the system with the predicted simulation given by the forward model. The search continues until the global or local minimum of the objective function, defined by the differences between the measured and simulated values of state variable(s), is obtained. Several optimization algorithms have been proposed to solve inverse problems. They include the steepest descent scheme, conjugate gradient method, Newton's algorithm, Gauss-Newton method, global optimization technique, Simplex method, Levenberg-Marquardt algorithm, quasi-Newton methods, genetic algorithm, and Monte Carlo-Markov Chain (MCMC) method [28,29].

The task seems straightforward; just a matter of selecting an appropriate mathematical model and estimating its parameters via optimization algorithms. However, several conceptual and computational difficulties have made the implementation of inverse modeling more challenging: (1) judicious choice of a mathematical model (forward model) that is representative enough to simulate the behavior of biological systems, with sufficient accuracy, and at the same time allows interpretation of the results beyond pure parameter estimation; (2) the type and quality of input data is a crucial prerequisite for successful parameter optimization by inverse modeling. The data should provide enough information regarding the excitation-response relationship of the system and have reasonable scatter; (3) well-posedness of the inverse problem, which depends on the model structure, the quality and quantity of the input data, and the type of imposed initial and boundary conditions [27,30].

The goal of this study is to develop, apply, and evaluate a general purpose inverse modeling strategy for identifying biomolecule mass transport and binding rate parameters from the FRAP protocol, studying possible inter-correlations among the parameters, analyzing possible ill-posedness of the inverse problem, and proposing approaches to obtain unique estimates for biomolecule mass transport and binding rate parameters. This approach has several advantages over direct measurement of parameters and commonly-used model calibration procedures. Unlike direct methods, inverse modeling does not impose any constraints on the form or complexity of the forward model, on the choice of initial and boundary conditions, on the constitutive relationships, or on the treatment of heterogeneities via deterministic or stochastic formulations. Therefore, experimental conditions can be chosen on the basis of convenience rather than by a need to simplify the mathematics of the processes. Additionally, if information regarding parameter uncertainty and model accuracy is needed, it can be obtained from the parameter optimization procedure.

The first section of this paper presents the mathematical model used to describe diffusion-reaction of biomolecules inside cells during the course of the FRAP experiment, along with the numerical algorithm used to solve it and the approach developed for parameter estimation by nonlinear optimization. The experimental studies, in which both a real FRAP experiment and simulations are considered, are presented in the second section. Results of parameter estimation for four distinct optimization scenarios are presented and discussed in the third section. This is followed by a possible method for obtaining unique values for biomolecule mass transport and reaction rate parameters, posedness (stability and uniqueness) analysis of the inverse problem, and the conclusion of the study.

## Theoretical study

### Formulation of the forward problem

Using primary rate kinetics, one can describe the binding reactions between free biomolecule and vacant binding sites during the course of the FRAP experiment by [14,16,26]:

F+S⇌KdKaC                         (1)
 MathType@MTEF@5@5@+=feaafiart1ev1aaatCvAUfKttLearuWrP9MDH5MBPbIqV92AaeXatLxBI9gBamXvP5wqSXMqHnxAJn0BKvguHDwzZbqegyvzYrwyUfgarqqtubsr4rNCHbGeaGqiA8vkIkVAFgIELiFeLkFeLk=iY=Hhbbf9v8qqaqFr0xc9pk0xbba9q8WqFfeaY=biLkVcLq=JHqVepeea0=as0db9vqpepesP0xe9Fve9Fve9GapdbaqaaeGacaGaaiaabeqaamqadiabaaGcbaGaemOrayKaey4kaSIaem4uam1aa4GdaSqaaiabdUealnaaBaaameaacqWGHbqyaeqaaaWcbaGaem4saS0aaSbaaWqaaiabdsgaKbqabaaakiaawcCicaGL9gcacqWGdbWqcaaMc8UaaGPaVlaaykW7caaMc8UaaGPaVlaaykW7caaMc8UaaGPaVlaaykW7caaMc8UaaGPaVlaaykW7caaMc8UaaGPaVlaaykW7caaMc8UaaGPaVlaaykW7caaMc8UaaGPaVlaaykW7caaMc8UaaGPaVlaaykW7caaMc8UaeiikaGIaeGymaeJaeiykaKcaaa@72B9@

where *F *is concentration of free biomolecule, *S *is concentration of vacant binding sites, *C *is concentration of the bound complex (*C *= *FS*), *K*_*a *_is the free biomolecule-vacant binding site association rate coefficient (*T*^-1^), and *K*_*d *_is dissociation rate coefficient (*T*^-1^). The equation only describes the binding process and assumes uniform distribution of the binding sites. To describe diffusion and reaction of the macro-molecule inside the cell during the course of the FRAP protocol, one needs to incorporate diffusion in the mathematical model. This can be achieved by writing a set of three coupled nonlinear partial differential equations in a cylindrical coordinate system:

∂F∂t=DFrr∂2F∂r2+DFrr1r∂F∂r+DFθθ1r2∂2F∂θ2+DFzz∂2F∂z2−KaFS+KdC∂S∂t=DSrr∂2S∂r2+DSrr1r∂S∂r+DSθθ1r2∂2S∂θ2+DSzz∂2S∂z2−KaFS+KdC     (2)∂C∂t=DCrr∂2C∂r2+DCrr1r∂C∂r+DCθθ1r2∂2F∂θ2+DCzz∂2C∂z2+KaFS−KdC
 MathType@MTEF@5@5@+=feaafiart1ev1aaatCvAUfKttLearuWrP9MDH5MBPbIqV92AaeXatLxBI9gBaebbnrfifHhDYfgasaacH8akY=wiFfYdH8Gipec8Eeeu0xXdbba9frFj0=OqFfea0dXdd9vqai=hGuQ8kuc9pgc9s8qqaq=dirpe0xb9q8qiLsFr0=vr0=vr0dc8meaabaqaciaacaGaaeqabaqabeGadaaakqaabeqaamaalaaabaGaeyOaIyRaemOrayeabaGaeyOaIyRaemiDaqhaaiabg2da9iabdseaenaaBaaaleaacqWGgbGrcqWGYbGCcqWGYbGCaeqaaOWaaSaaaeaacqGHciITdaahaaWcbeqaaiabikdaYaaakiabdAeagbqaaiabgkGi2kabdkhaYnaaCaaaleqabaGaeGOmaidaaaaakiabgUcaRiabdseaenaaBaaaleaacqWGgbGrcqWGYbGCcqWGYbGCaeqaaOWaaSaaaeaacqaIXaqmaeaacqWGYbGCaaWaaSaaaeaacqGHciITcqWGgbGraeaacqGHciITcqWGYbGCaaGaey4kaSIaemiraq0aaSbaaSqaaiabdAeagHGaciab=H7aXjab=H7aXbqabaGcdaWcaaqaaiabigdaXaqaaiabdkhaYnaaCaaaleqabaGaeGOmaidaaaaakmaalaaabaGaeyOaIy7aaWbaaSqabeaacqaIYaGmaaGccqWGgbGraeaacqGHciITcqWF4oqCdaahaaWcbeqaaGqaaiab+jdaYaaaaaGccqGHRaWkcqWGebardaWgaaWcbaGaemOrayKaemOEaONaemOEaOhabeaakmaalaaabaGaeyOaIy7aaWbaaSqabeaacqaIYaGmaaGccqWGgbGraeaacqGHciITcqWG6bGEdaahaaWcbeqaaiab+jdaYaaaaaGccqGHsislcqWGlbWsdaWgaaWcbaGaemyyaegabeaakiabdAeagjabdofatjabgUcaRiabdUealnaaBaaaleaacqWGKbazaeqaaOGaem4qameabaWaaSaaaeaacqGHciITcqWGtbWuaeaacqGHciITcqWG0baDaaGaeyypa0Jaemiraq0aaSbaaSqaaiabdofatjabdkhaYjabdkhaYbqabaGcdaWcaaqaaiabgkGi2oaaCaaaleqabaGaeGOmaidaaOGaem4uamfabaGaeyOaIyRaemOCai3aaWbaaSqabeaacqaIYaGmaaaaaOGaey4kaSIaemiraq0aaSbaaSqaaiabdofatjabdkhaYjabdkhaYbqabaGcdaWcaaqaaiabigdaXaqaaiabdkhaYbaadaWcaaqaaiabgkGi2kabdofatbqaaiabgkGi2kabdkhaYbaacqGHRaWkcqWGebardaWgaaWcbaacbiGae03uamLae8hUdeNae8hUdehabeaakmaalaaabaGaeGymaedabaGaemOCai3aaWbaaSqabeaacqaIYaGmaaaaaOWaaSaaaeaacqGHciITdaahaaWcbeqaaiabikdaYaaakiabdofatbqaaiabgkGi2kab=H7aXnaaCaaaleqabaGae4NmaidaaaaakiabgUcaRiabdseaenaaBaaaleaacqWGtbWucqWG6bGEcqWG6bGEaeqaaOWaaSaaaeaacqGHciITdaahaaWcbeqaaiabikdaYaaakiabdofatbqaaiabgkGi2kabdQha6naaCaaaleqabaGae4NmaidaaaaakiabgkHiTiabdUealnaaBaaaleaacqWGHbqyaeqaaOGaemOrayKaem4uamLaey4kaSIaem4saS0aaSbaaSqaaiabdsgaKbqabaGccqWGdbWqcaWLjaGaaCzcaiabcIcaOiabikdaYiabcMcaPaqaamaalaaabaGaeyOaIyRaem4qameabaGaeyOaIyRaemiDaqhaaiabg2da9iabdseaenaaBaaaleaacqWGdbWqcqWGYbGCcqWGYbGCaeqaaOWaaSaaaeaacqGHciITdaahaaWcbeqaaiabikdaYaaakiabdoeadbqaaiabgkGi2kabdkhaYnaaCaaaleqabaGaeGOmaidaaaaakiabgUcaRiabdseaenaaBaaaleaacqWGdbWqcqWGYbGCcqWGYbGCaeqaaOWaaSaaaeaacqaIXaqmaeaacqWGYbGCaaWaaSaaaeaacqGHciITcqWGdbWqaeaacqGHciITcqWGYbGCaaGaey4kaSIaemiraq0aaSbaaSqaaiab9neadjab=H7aXjab=H7aXbqabaGcdaWcaaqaaiabigdaXaqaaiabdkhaYnaaCaaaleqabaGae4NmaidaaaaakmaalaaabaGaeyOaIy7aaWbaaSqabeaacqaIYaGmaaGccqWGgbGraeaacqGHciITcqWF4oqCdaahaaWcbeqaaiab+jdaYaaaaaGccqGHRaWkcqWGebardaWgaaWcbaGaem4qamKaemOEaONaemOEaOhabeaakmaalaaabaGaeyOaIy7aaWbaaSqabeaacqaIYaGmaaGccqWGdbWqaeaacqGHciITcqWG6bGEdaahaaWcbeqaaiab+jdaYaaaaaGccqGHRaWkcqWGlbWsdaWgaaWcbaGaemyyaegabeaakiabdAeagjabdofatjabgkHiTiabdUealnaaBaaaleaacqWGKbazaeqaaOGaem4qameaaaa@1455@

in which *r *is radial coordinate (*L*) in the cylindrical coordinate system, and *D*_*F*_, *D*_*S*_, and *D*_*C *_are molecular diffusion coefficients (*L*^2^*T*^-1^) for free biomolecules, vacant binding sites, and bound complex, respectively (symbols *L *and *T *inside parentheses are dimensions).

To develop and solve equation (2) the following assumption were made:

1. The medium is isotropic and homogeneous and the axes of the diffusion tensors are parallel to those of the coordinate system. By these assumptions, the second-order diffusion tensors collapse to the diffusion coefficients *D*_*F*_, *D*_*S*_, and *D*_*C*_.

2. Two-dimensional diffusion takes place in the plane of focus. This is a legitimate assumption when the bleaching area creates a cylindrical path through the cell, which is the case for a circular bleach spot with reasonable spot size [14,16]. This assumption eliminates the azimuthal and vertical components of the coordinate system.

3. There are no advective velocity fields in the bleached area. We acknowledge that ignoring the convective flux will lead to the overestimation of the diffusion coefficient, but in the presence of a binding reaction this overestimation is negligible. In other words, we assume that the Peclet number is less than unity and advection is not dominant.

4. The effects of heating (caused by the absorption of the laser beam by the sample and fluorophore) on the biomolecule mass transport and binding rate parameters are negligible. In other words, we assume isothermal flow of biomolecules toward the bleached area from the undisturbed region.

5. The diffusion of the bound complex is negligible (*D*_*C *_= 0, *D*_*S *_= 0).

6. The biological system is in a state of equilibrium before photobleaching and it remains so over the time course of the FRAP experiment. This is a reasonable assumption because most biological FRAP experiments take from several seconds to several minutes, whereas the GFP-fusion expression changes over a time course of hours [14]. This eliminates the second equation in the system of three coupled nonlinear partial differential equations and hence Eq. (2) collapses to one site-mobile-immobile model:

∂F∂t=DF∂2F∂r2+DF1r∂F∂r−Ka*F+KdC∂C∂t=Ka*F−KdC     (3)
 MathType@MTEF@5@5@+=feaafiart1ev1aaatCvAUfKttLearuWrP9MDH5MBPbIqV92AaeXatLxBI9gBaebbnrfifHhDYfgasaacH8akY=wiFfYdH8Gipec8Eeeu0xXdbba9frFj0=OqFfea0dXdd9vqai=hGuQ8kuc9pgc9s8qqaq=dirpe0xb9q8qiLsFr0=vr0=vr0dc8meaabaqaciaacaGaaeqabaqabeGadaaakeaafaqaaeGabaaabaWaaSaaaeaacqGHciITcqWGgbGraeaacqGHciITcqWG0baDaaGaeyypa0Jaemiraq0aaSbaaSqaaiabdAeagbqabaGcdaWcaaqaaiabgkGi2oaaCaaaleqabaGaeGOmaidaaOGaemOrayeabaGaeyOaIyRaemOCai3aaWbaaSqabeaacqaIYaGmaaaaaOGaey4kaSIaemiraq0aaSbaaSqaaiabdAeagbqabaGcdaWcaaqaaiabigdaXaqaaiabdkhaYbaadaWcaaqaaiabgkGi2kabdAeagbqaaiabgkGi2kabdkhaYbaacqGHsislcqWGlbWsdaqhaaWcbaGaemyyaegabaGaeiOkaOcaaOGaemOrayKaey4kaSIaem4saS0aaSbaaSqaaiabdsgaKbqabaGccqWGdbWqaeaadaWcaaqaaiabgkGi2kabdoeadbqaaiabgkGi2kabdsha0baacqGH9aqpcqWGlbWsdaqhaaWcbaGaemyyaegabaGaeiOkaOcaaOGaemOrayKaeyOeI0Iaem4saS0aaSbaaSqaaiabdsgaKbqabaGccqWGdbWqaaGaaCzcaiaaxMaadaqadaqaaiabiodaZaGaayjkaiaawMcaaaaa@65A2@

Where Ka*
 MathType@MTEF@5@5@+=feaafiart1ev1aaatCvAUfKttLearuWrP9MDH5MBPbIqV92AaeXatLxBI9gBaebbnrfifHhDYfgasaacH8akY=wiFfYdH8Gipec8Eeeu0xXdbba9frFj0=OqFfea0dXdd9vqai=hGuQ8kuc9pgc9s8qqaq=dirpe0xb9q8qiLsFr0=vr0=vr0dc8meaabaqaciaacaGaaeqabaqabeGadaaakeaacqWGlbWsdaqhaaWcbaGaemyyaegabaGaeiOkaOcaaaaa@301F@ = *K*_*a*_*S *is the pseudo-association rate coefficient.

System (3) was solved analytically in Laplace space involving Bessel functions [14] for total fluorescence recovery averaged over the bleach spot (of radius *w*). The solution was adopted from that for a problem of heat conduction between two concentric cylinders [31]:

frap¯(s)=1s−Feqs[1−2K1(qw)I1(qw)][1+Ka*s+Kd]−Ceqs+Kd     (4)
 MathType@MTEF@5@5@+=feaafiart1ev1aaatCvAUfKttLearuWrP9MDH5MBPbIqV92AaeXatLxBI9gBaebbnrfifHhDYfgasaacH8akY=wiFfYdH8Gipec8Eeeu0xXdbba9frFj0=OqFfea0dXdd9vqai=hGuQ8kuc9pgc9s8qqaq=dirpe0xb9q8qiLsFr0=vr0=vr0dc8meaabaqaciaacaGaaeqabaqabeGadaaakeaadaqdaaqaaiabdAgaMjabdkhaYjabdggaHjabdchaWbaacqGGOaakcqWGZbWCcqGGPaqkcqGH9aqpdaWcaaqaaiabigdaXaqaaiabdohaZbaacqGHsisldaWcaaqaaiabdAeagnaaBaaaleaacqWGLbqzcqWGXbqCaeqaaaGcbaGaem4CamhaaiabcUfaBjabigdaXiabgkHiTiabikdaYiabdUealnaaBaaaleaacqaIXaqmaeqaaOWaaeWaaeaacqWGXbqCcqWG3bWDaiaawIcacaGLPaaacqWGjbqsdaWgaaWcbaGaeGymaedabeaakmaabmaabaGaemyCaeNaem4DaChacaGLOaGaayzkaaGaeiyxa0Laei4waSLaeGymaeJaey4kaSYaaSaaaeaacqWGlbWsdaqhaaWcbaGaemyyaegabaGaeiOkaOcaaaGcbaGaem4CamNaey4kaSIaem4saS0aaSbaaSqaaiabdsgaKbqabaaaaOGaeiyxa0LaeyOeI0YaaSaaaeaacqWGdbWqdaWgaaWcbaGaemyzauMaemyCaehabeaaaOqaaiabdohaZjabgUcaRiabdUealnaaBaaaleaacqWGKbazaeqaaaaakiaaxMaacaWLjaGaeiikaGIaeGinaqJaeiykaKcaaa@6CA2@

where:

q2=sDf[1+Ka*s+Kd]     (5)
 MathType@MTEF@5@5@+=feaafiart1ev1aaatCvAUfKttLearuWrP9MDH5MBPbIqV92AaeXatLxBI9gBaebbnrfifHhDYfgasaacH8akY=wiFfYdH8Gipec8Eeeu0xXdbba9frFj0=OqFfea0dXdd9vqai=hGuQ8kuc9pgc9s8qqaq=dirpe0xb9q8qiLsFr0=vr0=vr0dc8meaabaqaciaacaGaaeqabaqabeGadaaakeaacqWGXbqCdaahaaWcbeqaaiabikdaYaaakiabg2da9maalaaabaGaem4CamhabaGaemiraq0aaSbaaSqaaiabdAgaMbqabaaaaOGaei4waSLaeGymaeJaey4kaSYaaSaaaeaacqWGlbWsdaqhaaWcbaGaemyyaegabaGaeiOkaOcaaaGcbaGaem4CamNaey4kaSIaem4saS0aaSbaaSqaaiabdsgaKbqabaaaaOGaeiyxa0LaaCzcaiaaxMaacqGGOaakcqaI1aqncqGGPaqkaaa@4525@

Ceq=Ka*Ka*+Kd     (6)
 MathType@MTEF@5@5@+=feaafiart1ev1aaatCvAUfKttLearuWrP9MDH5MBPbIqV92AaeXatLxBI9gBaebbnrfifHhDYfgasaacH8akY=wiFfYdH8Gipec8Eeeu0xXdbba9frFj0=OqFfea0dXdd9vqai=hGuQ8kuc9pgc9s8qqaq=dirpe0xb9q8qiLsFr0=vr0=vr0dc8meaabaqaciaacaGaaeqabaqabeGadaaakeaacqWGdbWqdaWgaaWcbaGaemyzauMaemyCaehabeaakiabg2da9maalaaabaGaem4saS0aa0baaSqaaiabdggaHbqaaiabcQcaQaaaaOqaaiabdUealnaaDaaaleaacqWGHbqyaeaacqGGQaGkaaGccqGHRaWkcqWGlbWsdaWgaaWcbaGaemizaqgabeaaaaGccaWLjaGaaCzcaiabcIcaOiabiAda2iabcMcaPaaa@4037@

Feq=KdKa*+Kd     (7)
 MathType@MTEF@5@5@+=feaafiart1ev1aaatCvAUfKttLearuWrP9MDH5MBPbIqV92AaeXatLxBI9gBaebbnrfifHhDYfgasaacH8akY=wiFfYdH8Gipec8Eeeu0xXdbba9frFj0=OqFfea0dXdd9vqai=hGuQ8kuc9pgc9s8qqaq=dirpe0xb9q8qiLsFr0=vr0=vr0dc8meaabaqaciaacaGaaeqabaqabeGadaaakeaacqWGgbGrdaWgaaWcbaGaemyzauMaemyCaehabeaakiabg2da9maalaaabaGaem4saS0aaSbaaSqaaiabdsgaKbqabaaakeaacqWGlbWsdaqhaaWcbaGaemyyaegabaGaeiOkaOcaaOGaey4kaSIaem4saS0aaSbaaSqaaiabdsgaKbqabaaaaOGaaCzcaiaaxMaacqGGOaakcqaI3aWncqGGPaqkaaa@3F68@

*C*_*eq *_+ *F*_*eq *_= 1     (8)

In these expressions, *s *is the Laplace transform variable that inverts to yield time, frap¯
 MathType@MTEF@5@5@+=feaafiart1ev1aaatCvAUfKttLearuWrP9MDH5MBPbIqV92AaeXatLxBI9gBaebbnrfifHhDYfgasaacH8akY=wiFfYdH8Gipec8Eeeu0xXdbba9frFj0=OqFfea0dXdd9vqai=hGuQ8kuc9pgc9s8qqaq=dirpe0xb9q8qiLsFr0=vr0=vr0dc8meaabaqaciaacaGaaeqabaqabeGadaaakeaadaqdaaqaaiabdAgaMjabdkhaYjabdggaHjabdchaWbaaaaa@3233@(*s*) is the average of the Laplace transform of the fluorescent intensity within the bleach spot, *F*_*eq *_and *C*_*eq *_are equilibrium concentration of *F *and *C*, and *I*_1 _and *K*_1 _are modified Bessel functions of the first and second kind.

To obtain frap¯
 MathType@MTEF@5@5@+=feaafiart1ev1aaatCvAUfKttLearuWrP9MDH5MBPbIqV92AaeXatLxBI9gBaebbnrfifHhDYfgasaacH8akY=wiFfYdH8Gipec8Eeeu0xXdbba9frFj0=OqFfea0dXdd9vqai=hGuQ8kuc9pgc9s8qqaq=dirpe0xb9q8qiLsFr0=vr0=vr0dc8meaabaqaciaacaGaaeqabaqabeGadaaakeaadaqdaaqaaiabdAgaMjabdkhaYjabdggaHjabdchaWbaaaaa@3233@(*s*) as a function of time in real space, one needs to calculate the inverse Laplace transform numerically. In the present study, the MATLAB routine *invlap.m *[32] was used for this task.

#### Numerical solution strategy

In this study, the forward model (Eq. 3) is solved using a fully implicit backward in time and central in space finite difference approximation. The choice of a numerical approach was made so that the inversion method could be readily extended to arbitrary initial and boundary conditions and domain geometry, and especially so that it could be extended to the system of equations (2) rather than just its simplified version in (3). The corresponding discretization of equation (3) is:

Fin+1−FinΔt=DFFi+1n+1−2Fin+1+Fi−1n+1(Δr)2+DFrFi+1n+1−Fi−1n+12Δr−Ka*Fin+1+KdCin+1Cin+1−CinΔt=Ka*Fin+1−KdCin+1     (9)
 MathType@MTEF@5@5@+=feaafiart1ev1aaatCvAUfKttLearuWrP9MDH5MBPbIqV92AaeXatLxBI9gBaebbnrfifHhDYfgasaacH8akY=wiFfYdH8Gipec8Eeeu0xXdbba9frFj0=OqFfea0dXdd9vqai=hGuQ8kuc9pgc9s8qqaq=dirpe0xb9q8qiLsFr0=vr0=vr0dc8meaabaqaciaacaGaaeqabaqabeGadaaakeaafaqaaeGabaaabaWaaSaaaeaacqWGgbGrdaqhaaWcbaGaemyAaKgabaGaemOBa4Maey4kaSIaeGymaedaaOGaeyOeI0IaemOray0aa0baaSqaaiabdMgaPbqaaiabd6gaUbaaaOqaaiabfs5aejabdsha0baacqGH9aqpcqWGebardaWgaaWcbaGaemOrayeabeaakmaalaaabaGaemOray0aa0baaSqaaiabdMgaPjabgUcaRiabigdaXaqaaiabd6gaUjabgUcaRiabigdaXaaakiabgkHiTiabikdaYiabdAeagnaaDaaaleaacqWGPbqAaeaacqWGUbGBcqGHRaWkcqaIXaqmaaGccqGHRaWkcqWGgbGrdaqhaaWcbaGaemyAaKMaeyOeI0IaeGymaedabaGaemOBa4Maey4kaSIaeGymaedaaaGcbaWaaeWaaeaacqqHuoarcqWGYbGCaiaawIcacaGLPaaadaahaaWcbeqaaiabikdaYaaaaaGccqGHRaWkdaWcaaqaaiabdseaenaaBaaaleaacqWGgbGraeqaaaGcbaGaemOCaihaamaalaaabaGaemOray0aa0baaSqaaiabdMgaPjabgUcaRiabigdaXaqaaiabd6gaUjabgUcaRiabigdaXaaakiabgkHiTiabdAeagnaaDaaaleaacqWGPbqAcqGHsislcqaIXaqmaeaacqWGUbGBcqGHRaWkcqaIXaqmaaaakeaacqaIYaGmcqqHuoarcqWGYbGCaaGaeyOeI0Iaem4saS0aa0baaSqaaiabdggaHbqaaiabcQcaQaaakiabdAeagnaaDaaaleaacqWGPbqAaeaacqWGUbGBcqGHRaWkcqaIXaqmaaGccqGHRaWkcqWGlbWsdaWgaaWcbaGaemizaqgabeaakiabdoeadnaaDaaaleaacqWGPbqAaeaacqWGUbGBcqGHRaWkcqaIXaqmaaaakeaadaWcaaqaaiabdoeadnaaDaaaleaacqWGPbqAaeaacqWGUbGBcqGHRaWkcqaIXaqmaaGccqGHsislcqWGdbWqdaqhaaWcbaGaemyAaKgabaGaemOBa4gaaaGcbaGaeuiLdqKaemiDaqhaaiabg2da9iabdUealnaaDaaaleaacqWGHbqyaeaacqGGQaGkaaGccqWGgbGrdaqhaaWcbaGaemyAaKgabaGaemOBa4Maey4kaSIaeGymaedaaOGaeyOeI0Iaem4saS0aaSbaaSqaaiabdsgaKbqabaGccqWGdbWqdaqhaaWcbaGaemyAaKgabaGaemOBa4Maey4kaSIaeGymaedaaaaakiaaxMaacaWLjaWaaeWaaeaacqaI5aqoaiaawIcacaGLPaaaaaa@AD52@

Where *n *is the time step and *i *denotes location in space. Rearranging Eq. (9) one obtains the following block tri-diagonal matrix equation suitable for solution by a linear algebraic solver:

[DFΔtΔr(12r−1Δr)]Fi−1n+1+[1+2DFΔt(Δr)2+Ka*Δt]Fin+1−[DFΔtΔr(12r−1Δr)]Fi+1n+1−KdCin+1=Fin[1+KdΔt]Cin+1−Ka*ΔtFin+1=Cin     (10)
 MathType@MTEF@5@5@+=feaafiart1ev1aaatCvAUfKttLearuWrP9MDH5MBPbIqV92AaeXatLxBI9gBaebbnrfifHhDYfgasaacH8akY=wiFfYdH8Gipec8Eeeu0xXdbba9frFj0=OqFfea0dXdd9vqai=hGuQ8kuc9pgc9s8qqaq=dirpe0xb9q8qiLsFr0=vr0=vr0dc8meaabaqaciaacaGaaeqabaqabeGadaaakeaafaqaaeWabaaabaGaei4waS1aaSaaaeaacqWGebardaWgaaWcbaGaemOrayeabeaakiabfs5aejabdsha0bqaaiabfs5aejabdkhaYbaacqGGOaakdaWcaaqaaiabigdaXaqaaiabikdaYiabdkhaYbaacqGHsisldaWcaaqaaiabigdaXaqaaiabfs5aejabdkhaYbaacqGGPaqkcqGGDbqxcqWGgbGrdaqhaaWcbaGaemyAaKMaeyOeI0IaeGymaedabaGaemOBa4Maey4kaSIaeGymaedaaOGaey4kaSIaei4waSLaeGymaeJaey4kaSYaaSaaaeaacqaIYaGmcqWGebardaWgaaWcbaGaemOrayeabeaakiabfs5aejabdsha0bqaamaabmaabaGaeuiLdqKaemOCaihacaGLOaGaayzkaaWaaWbaaSqabeaacqaIYaGmaaaaaOGaey4kaSIaem4saS0aa0baaSqaaiabdggaHbqaaiabcQcaQaaakiabfs5aejabdsha0jabc2faDjabdAeagnaaDaaaleaacqWGPbqAaeaacqWGUbGBcqGHRaWkcqaIXaqmaaGccqGHsislaeaacqGGBbWwdaWcaaqaaiabdseaenaaBaaaleaacqWGgbGraeqaaOGaeuiLdqKaemiDaqhabaGaeuiLdqKaemOCaihaaiabcIcaOmaalaaabaGaeGymaedabaGaeGOmaiJaemOCaihaaiabgkHiTmaalaaabaGaeGymaedabaGaeuiLdqKaemOCaihaaiabcMcaPiabc2faDjabdAeagnaaDaaaleaacqWGPbqAcqGHRaWkcqaIXaqmaeaacqWGUbGBcqGHRaWkcqaIXaqmaaGccqGHsislcqWGlbWsdaWgaaWcbaGaemizaqgabeaakiabdoeadnaaDaaaleaacqWGPbqAaeaacqWGUbGBcqGHRaWkcqaIXaqmaaGccqGH9aqpcqWGgbGrdaqhaaWcbaGaemyAaKgabaGaemOBa4gaaaGcbaGaei4waSLaeGymaeJaey4kaSIaem4saS0aaSbaaSqaaiabdsgaKbqabaGccqqHuoarcqWG0baDcqGGDbqxcqWGdbWqdaqhaaWcbaGaemyAaKgabaGaemOBa4Maey4kaSIaeGymaedaaOGaeyOeI0Iaem4saS0aa0baaSqaaiabdggaHbqaaiabcQcaQaaakiabfs5aejabdsha0jabdAeagnaaDaaaleaacqWGPbqAaeaacqWGUbGBcqGHRaWkcqaIXaqmaaGccqGH9aqpcqWGdbWqdaqhaaWcbaGaemyAaKgabaGaemOBa4gaaaaakiaaxMaacaWLjaGaeiikaGIaeGymaeJaeGimaaJaeiykaKcaaa@B905@

To solve equation (10) the following initial conditions were used:

F(0,r)={0,0<r≤wFeq,w<r≤RC(0,r)={0,0<r≤wCeq,w<r≤R
 MathType@MTEF@5@5@+=feaafiart1ev1aaatCvAUfKttLearuWrP9MDH5MBPbIqV92AaeXatLxBI9gBaebbnrfifHhDYfgasaacH8akY=wiFfYdH8Gipec8Eeeu0xXdbba9frFj0=OqFfea0dXdd9vqai=hGuQ8kuc9pgc9s8qqaq=dirpe0xb9q8qiLsFr0=vr0=vr0dc8meaabaqaciaacaGaaeqabaqabeGadaaakqaabeqaaiabdAeagnaabmaabaGaeGimaaJaeiilaWIaemOCaihacaGLOaGaayzkaaGaeyypa0ZaaiqaaqaabeqaaiabicdaWiabcYcaSiabicdaWiabgYda8iabdkhaYjabgsMiJkabdEha3bqaaiabdAeagnaaBaaaleaacqWGLbqzcqWGXbqCaeqaaOGaeiilaWIaem4DaCNaeyipaWJaemOCaiNaeyizImQaemOuaifaaiaawUhaaaqaaiabdoeadnaabmaabaGaeGimaaJaeiilaWIaemOCaihacaGLOaGaayzkaaGaeyypa0ZaaiqaaqaabeqaaiabicdaWiabcYcaSiabicdaWiabgYda8iabdkhaYjabgsMiJkabdEha3bqaaiabdoeadnaaBaaaleaacqWGLbqzcqWGXbqCaeqaaOGaeiilaWIaem4DaCNaeyipaWJaemOCaiNaeyizImQaemOuaifaaiaawUhaaaaaaa@64BF@

where *w *is the radius of the bleached area and *R *is the length of the spatial domain. The initial condition implies that the act of bleaching destroys the fluorescence tag on the biomolecules in the bleached area but does not change the concentrations of free biomolecule, bound complex, or vacant binding sites. The boundary conditions were formulated as:

∂F∂r|r=0=∂F∂r|r→∞=0∂C∂r|r=0=∂C∂r|r→∞=0
 MathType@MTEF@5@5@+=feaafiart1ev1aaatCvAUfKttLearuWrP9MDH5MBPbIqV92AaeXatLxBI9gBaebbnrfifHhDYfgasaacH8akY=wiFfYdH8Gipec8Eeeu0xXdbba9frFj0=OqFfea0dXdd9vqai=hGuQ8kuc9pgc9s8qqaq=dirpe0xb9q8qiLsFr0=vr0=vr0dc8meaabaqaciaacaGaaeqabaqabeGadaaakqaabeqaamaaeiaabaWaaSaaaeaacqGHciITcqWGgbGraeaacqGHciITcqWGYbGCaaaacaGLiWoadaWgaaWcbaGaemOCaiNaeyypa0JaeGimaadabeaakiabg2da9maaeiaabaWaaSaaaeaacqGHciITcqWGgbGraeaacqGHciITcqWGYbGCaaaacaGLiWoadaWgaaWcbaGaemOCaiNaeyOKH4QaeyOhIukabeaakiabg2da9iabicdaWaqaamaaeiaabaWaaSaaaeaacqGHciITcqWGdbWqaeaacqGHciITcqWGYbGCaaaacaGLiWoadaWgaaWcbaGaemOCaiNaeyypa0JaeGimaadabeaakiabg2da9maaeiaabaWaaSaaaeaacqGHciITcqWGdbWqaeaacqGHciITcqWGYbGCaaaacaGLiWoadaWgaaWcbaGaemOCaiNaeyOKH4QaeyOhIukabeaakiabg2da9iabicdaWaaaaa@5F9B@

which imply that the diffusive biomolecule flux is zero at the center of the bleach spot and far beyond the bleached area throughout the course of the FRAP experiment.

This numerical solution was validated by comparing it to the analytical solution (4). For this purpose, the average of the fluorescence intensity within the bleach spot was calculated by [27]:

frap¯(s)=2w2∫0wr[F(r)+C(r)]dr     (11)
 MathType@MTEF@5@5@+=feaafiart1ev1aaatCvAUfKttLearuWrP9MDH5MBPbIqV92AaeXatLxBI9gBaebbnrfifHhDYfgasaacH8akY=wiFfYdH8Gipec8Eeeu0xXdbba9frFj0=OqFfea0dXdd9vqai=hGuQ8kuc9pgc9s8qqaq=dirpe0xb9q8qiLsFr0=vr0=vr0dc8meaabaqaciaacaGaaeqabaqabeGadaaakeaadaqdaaqaaiabdAgaMjabdkhaYjabdggaHjabdchaWbaadaqadaqaaiabdohaZbGaayjkaiaawMcaaiabg2da9maalaaabaGaeGOmaidabaGaem4DaC3aaWbaaSqabeaacqaIYaGmaaaaaOWaa8qmaeaacqWGYbGCcqGGBbWwcqWGgbGrdaqadaqaaiabdkhaYbGaayjkaiaawMcaaaWcbaGaeGimaadabaGaem4DaChaniabgUIiYdGccqGHRaWkcqWGdbWqdaqadaqaaiabdkhaYbGaayjkaiaawMcaaiabc2faDjabdsgaKjabdkhaYjaaxMaacaWLjaGaeiikaGIaeGymaeJaeGymaeJaeiykaKcaaa@52DE@

**Figure 1:**
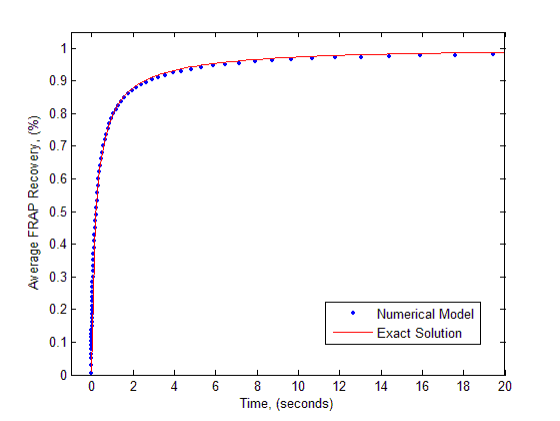
Validation of the numerical model with analytical solution. Parameter values *D*_*f *_= l.3 *μm*^2 ^*s*^-1^, Ka*
 MathType@MTEF@5@5@+=feaafiart1ev1aaatCvAUfKttLearuWrP9MDH5MBPbIqV92AaeXatLxBI9gBaebbnrfifHhDYfgasaacH8akY=wiFfYdH8Gipec8Eeeu0xXdbba9frFj0=OqFfea0dXdd9vqai=hGuQ8kuc9pgc9s8qqaq=dirpe0xb9q8qiLsFr0=vr0=vr0dc8meaabaqaciaacaGaaeqabaqabeGadaaakeaacqWGlbWsdaqhaaWcbaGaemyyaegabaGaeiOkaOcaaaaa@301F@ = 0.01*s*^-1^, *K*_*d *_= 0.25 *s*^-1^, and *w *= 0.5 *μm *were used to generate the graph in both solutions.

The results of the comparison for typical parameter values of *D*_*f *_= 1.3 *μm*^2 ^*s*^-1^, Ka*
 MathType@MTEF@5@5@+=feaafiart1ev1aaatCvAUfKttLearuWrP9MDH5MBPbIqV92AaeXatLxBI9gBaebbnrfifHhDYfgasaacH8akY=wiFfYdH8Gipec8Eeeu0xXdbba9frFj0=OqFfea0dXdd9vqai=hGuQ8kuc9pgc9s8qqaq=dirpe0xb9q8qiLsFr0=vr0=vr0dc8meaabaqaciaacaGaaeqabaqabeGadaaakeaacqWGlbWsdaqhaaWcbaGaemyyaegabaGaeiOkaOcaaaaa@301F@ = 0.01*s*^-1^, *K*_*d *_= 0.25*s*^-1^, and *w *= 0.5 *μm *are presented in Figure 1. These results confirm that the numerical approach used in this study does indeed produce an accurate solution of Eq. (3).

### Formulation of the inverse problem

We want to solve the unconstrained minimization problem (see Appendix for detailed derivation of equation (12)):

min⁡φ(p)=12∑i=1Nri(p)2=12r(p)Tr(p)     (12)
 MathType@MTEF@5@5@+=feaafiart1ev1aaatCvAUfKttLearuWrP9MDH5MBPbIqV92AaeXatLxBI9gBaebbnrfifHhDYfgasaacH8akY=wiFfYdH8Gipec8Eeeu0xXdbba9frFj0=OqFfea0dXdd9vqai=hGuQ8kuc9pgc9s8qqaq=dirpe0xb9q8qiLsFr0=vr0=vr0dc8meaabaqaciaacaGaaeqabaqabeGadaaakeaacyGGTbqBcqGGPbqAcqGGUbGBiiGacqWFgpGzdaqadaqaaGqaciab+bhaWbGaayjkaiaawMcaaiabg2da9maalaaabaGaeGymaedabaGaeGOmaidaamaaqahabaGaemOCai3aaSbaaSqaaiabdMgaPbqabaGcdaqadaqaaiabdchaWbGaayjkaiaawMcaamaaCaaaleqabaGaeGOmaidaaaqaaiabdMgaPjabg2da9iabigdaXaqaaiabd6eaobqdcqGHris5aOGaeyypa0ZaaSaaaeaacqaIXaqmaeaacqaIYaGmaaGaemOCai3aaeWaaeaacqWGWbaCaiaawIcacaGLPaaadaahaaWcbeqaaiabdsfaubaakiabdkhaYnaabmaabaGaemiCaahacaGLOaGaayzkaaGaaCzcaiaaxMaacqGGOaakcqaIXaqmcqaIYaGmcqGGPaqkaaa@583A@

where *r *is the residual (differences between the observed and predicted state variable) column vector, *N *is the number of observations, and 12
 MathType@MTEF@5@5@+=feaafiart1ev1aaatCvAUfKttLearuWrP9MDH5MBPbIqV92AaeXatLxBI9gBaebbnrfifHhDYfgasaacH8akY=wiFfYdH8Gipec8Eeeu0xXdbba9frFj0=OqFfea0dXdd9vqai=hGuQ8kuc9pgc9s8qqaq=dirpe0xb9q8qiLsFr0=vr0=vr0dc8meaabaqaciaacaGaaeqabaqabeGadaaakeaadaWcaaqaaiabigdaXaqaaiabikdaYaaaaaa@2E9E@ is only for notational convenience. Assuming *φ*(*p*) is twice-continuously differentiable, the gradient vector, ∇*φ*(*p*), and the Hessian matrix, ∇^2^*φ*(*p*), of *φ*(*p*) can be calculated as [33]:

∇φ(p)=∑i=1Nr1(p)∂ri(p)∂pi=−J(p)Tr(p)     (13)
 MathType@MTEF@5@5@+=feaafiart1ev1aaatCvAUfKttLearuWrP9MDH5MBPbIqV92AaeXatLxBI9gBaebbnrfifHhDYfgasaacH8akY=wiFfYdH8Gipec8Eeeu0xXdbba9frFj0=OqFfea0dXdd9vqai=hGuQ8kuc9pgc9s8qqaq=dirpe0xb9q8qiLsFr0=vr0=vr0dc8meaabaqaciaacaGaaeqabaqabeGadaaakeaacqGHhis0iiGacqWFgpGzdaqadaqaaiabdchaWbGaayjkaiaawMcaaiabg2da9maaqahabaGaemOCai3aaSbaaSqaaiabigdaXaqabaGcdaqadaqaaiabdchaWbGaayjkaiaawMcaamaalaaabaGaeyOaIyRaemOCai3aaSbaaSqaaiabdMgaPbqabaGcdaqadaqaaiabdchaWbGaayjkaiaawMcaaaqaaiabgkGi2kabdchaWnaaBaaaleaacqWGPbqAaeqaaaaakiabg2da9iabgkHiTiabdQeaknaabmaabaGaemiCaahacaGLOaGaayzkaaWaaWbaaSqabeaacqWGubavaaGccqWGYbGCdaqadaqaaiabdchaWbGaayjkaiaawMcaaiaaxMaacaWLjaGaeiikaGIaeGymaeJaeG4mamJaeiykaKcaleaacqWGPbqAcqGH9aqpcqaIXaqmaeaacqWGobGta0GaeyyeIuoaaaa@5C90@

∇2φ(p)=∑i=1N[∂ri(p)∂pj∂ri(p)∂pi+∂ri2(p)∂pi∂pjri(p)]=J(p)TJ(p)+∑i=1N∂ri2(p)∂pi∂pjri(p)     (14)
 MathType@MTEF@5@5@+=feaafiart1ev1aaatCvAUfKttLearuWrP9MDH5MBPbIqV92AaeXatLxBI9gBaebbnrfifHhDYfgasaacH8akY=wiFfYdH8Gipec8Eeeu0xXdbba9frFj0=OqFfea0dXdd9vqai=hGuQ8kuc9pgc9s8qqaq=dirpe0xb9q8qiLsFr0=vr0=vr0dc8meaabaqaciaacaGaaeqabaqabeGadaaakeaacqGHhis0daahaaWcbeqaaiabikdaYaaaiiGakiab=z8aMnaabmaabaGaemiCaahacaGLOaGaayzkaaGaeyypa0ZaaabCaeaacqGGBbWwdaWcaaqaaiabgkGi2kabdkhaYnaaBaaaleaacqWGPbqAaeqaaOWaaeWaaeaacqWGWbaCaiaawIcacaGLPaaaaeaacqGHciITcqWGWbaCdaWgaaWcbaGaemOAaOgabeaaaaGcdaWcaaqaaiabgkGi2kabdkhaYnaaBaaaleaacqWGPbqAaeqaaOWaaeWaaeaacqWGWbaCaiaawIcacaGLPaaaaeaacqGHciITcqWGWbaCdaWgaaWcbaGaemyAaKgabeaaaaaabaGaemyAaKMaeyypa0JaeGymaedabaGaemOta4eaniabggHiLdGccqGHRaWkdaWcaaqaaiabgkGi2kabdkhaYnaaDaaaleaacqWGPbqAaeaacqaIYaGmaaGcdaqadaqaaiabdchaWbGaayjkaiaawMcaaaqaaiabgkGi2kabdchaWnaaBaaaleaacqWGPbqAaeqaaOGaeyOaIyRaemiCaa3aaSbaaSqaaiabdQgaQbqabaaaaOGaemOCai3aaSbaaSqaaiabdMgaPbqabaGcdaqadaqaaiabdchaWbGaayjkaiaawMcaaiabc2faDjabg2da9iabdQeaknaabmaabaGaemiCaahacaGLOaGaayzkaaWaaWbaaSqabeaacqWGubavaaGccqWGkbGsdaqadaqaaiabdchaWbGaayjkaiaawMcaaiabgUcaRmaaqahabaWaaSaaaeaacqGHciITcqWGYbGCdaqhaaWcbaGaemyAaKgabaGaeGOmaidaaOWaaeWaaeaacqWGWbaCaiaawIcacaGLPaaaaeaacqGHciITcqWGWbaCdaWgaaWcbaGaemyAaKgabeaakiabgkGi2kabdchaWnaaBaaaleaacqWGQbGAaeqaaaaaaeaacqWGPbqAcqGH9aqpcqaIXaqmaeaacqWGobGta0GaeyyeIuoakiabdkhaYnaaBaaaleaacqWGPbqAaeqaaOWaaeWaaeaacqWGWbaCaiaawIcacaGLPaaacaWLjaGaaCzcaiabcIcaOiabigdaXiabisda0iabcMcaPaaa@9BBD@

Owing to the nonlinear nature of Eq. (12), its minimization was carried out iteratively by first starting with an initial guess of parameter vector, {*p*^(*k*)^} and updating it at each iteration until the termination criteria were met:

*p*^(*k*+1) ^= *p*^(*k*) ^+ *α *^(*k*) ^Δ *p*^(*k*) ^    (15)

where *a*^(*k*) ^is a scalar step length and Δ*p*^(*k*) ^is the direction of search (step direction).

The linear least square problem below, which avoids the computation of possibly ill-conditioned *J*(*p*^(*k*)^)^*T*^*J*(*p*^(*k*)^) [34,35], was solved to obtain the search direction in each iteration:

min⁡‖(r(pk)0)+(J(p)k(λkDk)12)Δpk‖2     (16)
 MathType@MTEF@5@5@+=feaafiart1ev1aaatCvAUfKttLearuWrP9MDH5MBPbIqV92AaeXatLxBI9gBaebbnrfifHhDYfgasaacH8akY=wiFfYdH8Gipec8Eeeu0xXdbba9frFj0=OqFfea0dXdd9vqai=hGuQ8kuc9pgc9s8qqaq=dirpe0xb9q8qiLsFr0=vr0=vr0dc8meaabaqaciaacaGaaeqabaqabeGadaaakeaacyGGTbqBcqGGPbqAcqGGUbGBdaqbdaqaamaabmaaeaqabeaacqWGYbGCdaqadaqaaiabdchaWnaaCaaaleqabaGaem4AaSgaaaGccaGLOaGaayzkaaaabaGaeGimaadaaiaawIcacaGLPaaacqGHRaWkdaqadaabaeqabaGaemOsaO0aaeWaaeaacqWGWbaCaiaawIcacaGLPaaadaahaaWcbeqaaiabdUgaRbaaaOqaamaabmaabaGaeq4UdW2aaWbaaSqabeaacqWGRbWAaaGccqWGebardaahaaWcbeqaaiabdUgaRbaaaOGaayjkaiaawMcaamaaCaaaleqabaWaaSaaaeaacqaIXaqmaeaacqaIYaGmaaaaaaaakiaawIcacaGLPaaacqqHuoarcqWGWbaCdaahaaWcbeqaaiabdUgaRbaaaOGaayzcSlaawQa7amaaCaaaleqabaGaeGOmaidaaOGaaCzcaiaaxMaacqGGOaakcqaIXaqmcqaI2aGncqGGPaqkaaa@5890@

We used *QR *decomposition [36] to solve Eq. (16).

A combination of "one-sided" and "two-sided" finite difference methods [37,38] was used to calculate the partial derivatives of the state variable (frap¯
 MathType@MTEF@5@5@+=feaafiart1ev1aaatCvAUfKttLearuWrP9MDH5MBPbIqV92AaeXatLxBI9gBaebbnrfifHhDYfgasaacH8akY=wiFfYdH8Gipec8Eeeu0xXdbba9frFj0=OqFfea0dXdd9vqai=hGuQ8kuc9pgc9s8qqaq=dirpe0xb9q8qiLsFr0=vr0=vr0dc8meaabaqaciaacaGaaeqabaqabeGadaaakeaadaqdaaqaaiabdAgaMjabdkhaYjabdggaHjabdchaWbaaaaa@3233@(*s*)) with respect to model parameters and to construct the Jacobian matrix:

J=∂ri(p)∂pi=−∂frap¯(p)∂pi     (17)
 MathType@MTEF@5@5@+=feaafiart1ev1aaatCvAUfKttLearuWrP9MDH5MBPbIqV92AaeXatLxBI9gBaebbnrfifHhDYfgasaacH8akY=wiFfYdH8Gipec8Eeeu0xXdbba9frFj0=OqFfea0dXdd9vqai=hGuQ8kuc9pgc9s8qqaq=dirpe0xb9q8qiLsFr0=vr0=vr0dc8meaabaqaciaacaGaaeqabaqabeGadaaakeaacqWGkbGscqGH9aqpdaWcaaqaaiabgkGi2kabdkhaYnaaBaaaleaacqWGPbqAaeqaaOWaaeWaaeaacqWGWbaCaiaawIcacaGLPaaaaeaacqGHciITcqWGWbaCdaWgaaWcbaGaemyAaKgabeaaaaGccqGH9aqpcqGHsisldaWcaaqaaiabgkGi2oaanaaabaGaemOzayMaemOCaiNaemyyaeMaemiCaahaamaabmaabaGaemiCaahacaGLOaGaayzkaaaabaGaeyOaIyRaemiCaa3aaSbaaSqaaiabdMgaPbqabaaaaOGaaCzcaiaaxMaacqGGOaakcqaIXaqmcqaI3aWncqGGPaqkaaa@4FB9@

in each iteration.

At the early stages of the optimization, where the search is far from the solution, the "one-sided" finite difference scheme, which is computationally cheap, was used [39]:

J=−frap¯(p1,p2,....pi+Δpi,...pp)−frap¯(p1,p2,....pi,...pp)Δpi     (18)
 MathType@MTEF@5@5@+=feaafiart1ev1aaatCvAUfKttLearuWrP9MDH5MBPbIqV92AaeXatLxBI9gBaebbnrfifHhDYfgasaacH8akY=wiFfYdH8Gipec8Eeeu0xXdbba9frFj0=OqFfea0dXdd9vqai=hGuQ8kuc9pgc9s8qqaq=dirpe0xb9q8qiLsFr0=vr0=vr0dc8meaabaqaciaacaGaaeqabaqabeGadaaakeaacqWGkbGscqGH9aqpcqGHsisldaWcaaqaamaanaaabaGaemOzayMaemOCaiNaemyyaeMaemiCaahaamaabmaabaGaemiCaa3aaSbaaSqaaiabigdaXaqabaGccqGGSaalcqWGWbaCdaWgaaWcbaGaeGOmaidabeaakiabcYcaSiabc6caUiabc6caUiabc6caUiabc6caUiabdchaWnaaBaaaleaacqWGPbqAaeqaaOGaey4kaSIaeuiLdqKaemiCaa3aaSbaaSqaaiabdMgaPbqabaGccqGGSaalcqGGUaGlcqGGUaGlcqGGUaGlcqWGWbaCdaWgaaWcbaGaemiCaahabeaaaOGaayjkaiaawMcaaiabgkHiTmaanaaabaGaemOzayMaemOCaiNaemyyaeMaemiCaahaamaabmaabaGaemiCaa3aaSbaaSqaaiabigdaXaqabaGccqGGSaalcqWGWbaCdaWgaaWcbaGaeGOmaidabeaakiabcYcaSiabc6caUiabc6caUiabc6caUiabc6caUiabdchaWnaaBaaaleaacqWGPbqAaeqaaOGaeiilaWIaeiOla4IaeiOla4IaeiOla4IaemiCaa3aaSbaaSqaaiabdchaWbqabaaakiaawIcacaGLPaaaaeaacqqHuoarcqWGWbaCdaWgaaWcbaGaemyAaKgabeaaaaGccaWLjaGaaCzcaiabcIcaOiabigdaXiabiIda4iabcMcaPaaa@755B@

As the optimization proceeds in descent direction, the algorithm switches to a more accurate but computationally expensive approach in which the partial derivatives of frap¯
 MathType@MTEF@5@5@+=feaafiart1ev1aaatCvAUfKttLearuWrP9MDH5MBPbIqV92AaeXatLxBI9gBaebbnrfifHhDYfgasaacH8akY=wiFfYdH8Gipec8Eeeu0xXdbba9frFj0=OqFfea0dXdd9vqai=hGuQ8kuc9pgc9s8qqaq=dirpe0xb9q8qiLsFr0=vr0=vr0dc8meaabaqaciaacaGaaeqabaqabeGadaaakeaadaqdaaqaaiabdAgaMjabdkhaYjabdggaHjabdchaWbaaaaa@3233@(*s*) with respect to the model parameters are calculated using a two-sided finite difference scheme:

J=−frap¯(p1,p2,....pi+Δpi,...pp)−frap¯(p1,p2,....pi−Δpi,...pp)2Δpi     (19)
 MathType@MTEF@5@5@+=feaafiart1ev1aaatCvAUfKttLearuWrP9MDH5MBPbIqV92AaeXatLxBI9gBaebbnrfifHhDYfgasaacH8akY=wiFfYdH8Gipec8Eeeu0xXdbba9frFj0=OqFfea0dXdd9vqai=hGuQ8kuc9pgc9s8qqaq=dirpe0xb9q8qiLsFr0=vr0=vr0dc8meaabaqaciaacaGaaeqabaqabeGadaaakeaacqWGkbGscqGH9aqpcqGHsisldaWcaaqaamaanaaabaGaemOzayMaemOCaiNaemyyaeMaemiCaahaamaabmaabaGaemiCaa3aaSbaaSqaaiabigdaXaqabaGccqGGSaalcqWGWbaCdaWgaaWcbaGaeGOmaidabeaakiabcYcaSiabc6caUiabc6caUiabc6caUiabc6caUiabdchaWnaaBaaaleaacqWGPbqAaeqaaOGaey4kaSIaeuiLdqKaemiCaa3aaSbaaSqaaiabdMgaPbqabaGccqGGSaalcqGGUaGlcqGGUaGlcqGGUaGlcqWGWbaCdaWgaaWcbaGaemiCaahabeaaaOGaayjkaiaawMcaaiabgkHiTmaanaaabaGaemOzayMaemOCaiNaemyyaeMaemiCaahaamaabmaabaGaemiCaa3aaSbaaSqaaiabigdaXaqabaGccqGGSaalcqWGWbaCdaWgaaWcbaGaeGOmaidabeaakiabcYcaSiabc6caUiabc6caUiabc6caUiabc6caUiabdchaWnaaBaaaleaacqWGPbqAaeqaaOGaeyOeI0IaeuiLdqKaemiCaa3aaSbaaSqaaiabdMgaPbqabaGccqGGSaalcqGGUaGlcqGGUaGlcqGGUaGlcqWGWbaCdaWgaaWcbaGaemiCaahabeaaaOGaayjkaiaawMcaaaqaaiabikdaYiabfs5aejabdchaWnaaBaaaleaacqWGPbqAaeqaaaaakiaaxMaacaWLjaGaeiikaGIaeGymaeJaeGyoaKJaeiykaKcaaa@7B9C@

The switch is made when *φ*(*p*) ≤ 1 × 10^-2^. A detailed description of the procedure to update the Jacobian matrix is presented in [39].

To ensure positive-definiteness of the Hessian matrix and the descent property of the algorithm, the value of *D *was initialized using a *p *× *p *identity matrix before the beginning of the optimization process. Then the diagonal elements were updated in each iteration as follows [27,39];

dj0=‖Jj0‖dji=max⁡(djk−1,‖Jjk‖)
 MathType@MTEF@5@5@+=feaafiart1ev1aaatCvAUfKttLearuWrP9MDH5MBPbIqV92AaeXatLxBI9gBaebbnrfifHhDYfgasaacH8akY=wiFfYdH8Gipec8Eeeu0xXdbba9frFj0=OqFfea0dXdd9vqai=hGuQ8kuc9pgc9s8qqaq=dirpe0xb9q8qiLsFr0=vr0=vr0dc8meaabaqaciaacaGaaeqabaqabeGadaaakqaabeqaaiabdsgaKnaaDaaaleaacqWGQbGAaeaacqaIWaamaaGccqGH9aqpdaqbdaqaaiabdQeaknaaDaaaleaacqWGQbGAaeaacqaIWaamaaaakiaawMa7caGLkWoaaeaacqWGKbazdaqhaaWcbaGaemOAaOgabaGaemyAaKgaaOGaeyypa0JagiyBa0MaeiyyaeMaeiiEaGNaeiikaGIaemizaq2aa0baaSqaaiabdQgaQbqaaiabdUgaRjabgkHiTiabigdaXaaakiabcYcaSmaafmaabaGaemOsaO0aa0baaSqaaiabdQgaQbqaaiabdUgaRbaaaOGaayzcSlaawQa7aiabcMcaPaaaaa@51A8@

where *j *is the *j*^*th *^column of the Jacobian matrix and *k *is the iteration level in the inverse algorithm. The lines below were implemented in the algorithm to update *D *at each iteration:

*for i *= 1: *p*

   *D*(*i*, *i*) = max (*norm*(*J*(:, *i*), *D*(*i*, *i*)))

end

In order to update *λ *at each iteration, the optimization starts with an initial parameter vector and a large *λ *(*λ *= 1). As long as the objective function decreases in each iteration, the value of *λ *is reduced. Otherwise, it is increased. The approach avoids calculation of *λ *and step length in each iteration and is therefore computationally cheap. A detailed description of the code for updating *λ *is given in [33].

Finally, to stop the algorithm and to end the search, a combined termination criterion was used (see [39] for detailed discussion):

if(∇φ(p)p=p^≤1×10−3&Δφ(p)φ(p)≤1×10−6&φ(p)≤1×10−2)
 MathType@MTEF@5@5@+=feaafiart1ev1aaatCvAUfKttLearuWrP9MDH5MBPbIqV92AaeXatLxBI9gBaebbnrfifHhDYfgasaacH8akY=wiFfYdH8Gipec8Eeeu0xXdbba9frFj0=OqFfea0dXdd9vqai=hGuQ8kuc9pgc9s8qqaq=dirpe0xb9q8qiLsFr0=vr0=vr0dc8meaabaqaciaacaGaaeqabaqabeGadaaakeGabaatfiabdMgaPjabdAgaMjabcIcaOiabgEGirJGaciab=z8aMnaabmaabaGaemiCaahacaGLOaGaayzkaaWaaSbaaSqaaiabdchaWjabg2da9iqbdchaWzaajaaabeaakiabgsMiJkabigdaXiabgEna0kabigdaXiabicdaWmaaCaaaleqabaGaeyOeI0IaeG4mamdaaOGaeiOjayYaaSaaaeaacqqHuoarcqWFgpGzdaqadaqaaiabdchaWbGaayjkaiaawMcaaaqaaiab=z8aMnaabmaabaGaemiCaahacaGLOaGaayzkaaaaaiabgsMiJkabigdaXiabgEna0kabigdaXiabicdaWmaaCaaaleqabaGaeyOeI0IaeGOnaydaaOGaeiOjayIae8NXdy2aaeWaaeaacqWGWbaCaiaawIcacaGLPaaacqGHKjYOcqaIXaqmcqGHxdaTcqaIXaqmcqaIWaamdaahaaWcbeqaaiabgkHiTiabikdaYaaakiabcMcaPaaa@66E2@

   *Stop*

else

   *Continue Optimization Loop*

end

The developed inverse modeling strategy was then used to quantify biomolecule mass transport and binding rate parameters.

## Experimental study

**Figure 2:**
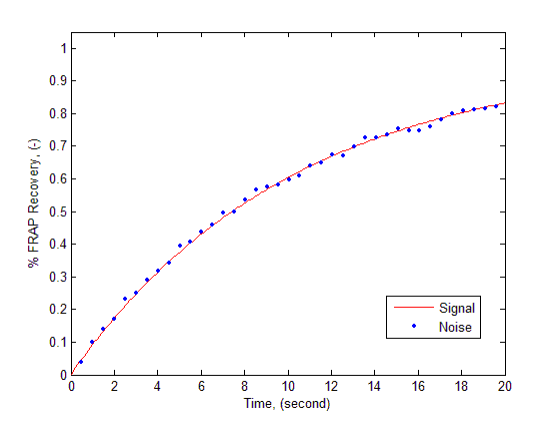
The generated noise free and noisy signals for FRAP protocol. The signal was generated by solving Eq. (3) for a hypothetical cell with prescribed initial and boundary conditions and parameter values: *D*_*f *_= 30 *μm*^2 ^*s*^-1^, Ka*
 MathType@MTEF@5@5@+=feaafiart1ev1aaatCvAUfKttLearuWrP9MDH5MBPbIqV92AaeXatLxBI9gBaebbnrfifHhDYfgasaacH8akY=wiFfYdH8Gipec8Eeeu0xXdbba9frFj0=OqFfea0dXdd9vqai=hGuQ8kuc9pgc9s8qqaq=dirpe0xb9q8qiLsFr0=vr0=vr0dc8meaabaqaciaacaGaaeqabaqabeGadaaakeaacqWGlbWsdaqhaaWcbaGaemyyaegabaGaeiOkaOcaaaaa@301F@ = 30 *s*^-1^, *K*_*d *_= 0.1108 *s*^-1^, and *w *= 0.5 *μm*.

To determine the mass transport and binding rate parameters of the GFP-tagged glucocorticoid receptor through the developed inverse modeling strategy, three data sets were used:

1. A FRAP experiment was conducted on the mouse adenocarcinoma cell line 3617 (McNally, personal communication), referred to as scenario A. This data set consists of 43 fluorescent recovery values gathered in the course of a 20-second FRAP experiment and post-processed to remove noise.

2. A generated data set was obtained by solving Eq. (3) for a hypothetical cell with prescribed initial and boundary conditions and parameter values: *D*_*f *_= 30 *μm*^2 ^*s*^-1^, Ka*
 MathType@MTEF@5@5@+=feaafiart1ev1aaatCvAUfKttLearuWrP9MDH5MBPbIqV92AaeXatLxBI9gBaebbnrfifHhDYfgasaacH8akY=wiFfYdH8Gipec8Eeeu0xXdbba9frFj0=OqFfea0dXdd9vqai=hGuQ8kuc9pgc9s8qqaq=dirpe0xb9q8qiLsFr0=vr0=vr0dc8meaabaqaciaacaGaaeqabaqabeGadaaakeaacqWGlbWsdaqhaaWcbaGaemyyaegabaGaeiOkaOcaaaaa@301F@ = 30*s*^-1^, *K*_*d *_= 0.1108*s*^-1^, and *w *= 0.5 *μm*. The reason for selecting these parameter values for data generation and parameter optimization is that they represent a situation in which the Damkohler number is almost unity and neither of the diffusion and reaction regimes is dominant. Both these processes are present in the experimental procedure. The parameter values also imply that the free GFP-GR molecules are mobile and the bound complex and the vacant binding sites are relatively immobile (*D*_*C *_= 0, *D*_*S *_= 0). Predicted FRAP recovery values were sampled at discrete times. The data were corrupted by adding normally distributed (*N*(0,0.01)) random error to each "measurement". The synthetic data were then used as input for parameter optimization problem and posedness analysis of the inverse problem. The resulting signal and noise are depicted in Figure 2.

3. The third data set was similar to the second but without perturbation. The data were used to determine what can and cannot be identified using FRAP data.

Four optimization scenarios were considered. In scenario A, the developed inverse modeling strategy was used to identify three unknown parameters [*D*_*f*_, *K*_*a*_, *K*_*d*_] for GFP-GR using the experimental FRAP data. To test the uniqueness of the model parameters, the optimization algorithm was carried out using different initial guesses for the parameter vector (*β *= [*D*_*f*_, Ka*
 MathType@MTEF@5@5@+=feaafiart1ev1aaatCvAUfKttLearuWrP9MDH5MBPbIqV92AaeXatLxBI9gBaebbnrfifHhDYfgasaacH8akY=wiFfYdH8Gipec8Eeeu0xXdbba9frFj0=OqFfea0dXdd9vqai=hGuQ8kuc9pgc9s8qqaq=dirpe0xb9q8qiLsFr0=vr0=vr0dc8meaabaqaciaacaGaaeqabaqabeGadaaakeaacqWGlbWsdaqhaaWcbaGaemyyaegabaGaeiOkaOcaaaaa@301F@, *K*_*d*_]). In scenario B, two of the three parameters in one-site-mobile-immobile model were kept constant and the third was estimated. The goal was to determine whether or not the FRAP protocol produces enough information to estimate one parameter uniquely. The optimization algorithm was used to estimate a single parameter for both noise-free and noisy data. In scenario C, pairs of model parameters were estimated under the assumption that the value of the third parameter is known. In the first attempt, the optimized values of the individual binding rate coefficients were quantified given a known value for the free molecular diffusion coefficient of the GFP-GR. Again the optimization algorithm was used for both noise-free and noisy data. Given the value of the pseudo-association rate, the optimized values of the molecular diffusion coefficient and dissociation rate coefficient were then estimated. Assuming that the "true" value of the dissociation rate coefficient is known, we tried to estimate the optimized values of the free molecular diffusion coefficient and the pseudo-association rate parameter. Again, the goal was to determine which pairs of parameters, if any, can be estimated uniquely using FRAP data. Finally, in scenario D, we investigated the possibility of simultaneous estimation of three parameters of the one-site-mobile-immobile model using noise-free FRAP data.

In all the scenarios investigated, the accuracy of the estimation was quantified by calculating and analyzing goodness-of-fit indices such Root Mean Squared Error (*RMSE*) and the Coefficient of Determination (*R*^2^). The root mean squared error and coefficient of determination were calculated as follows [27,40,41]:

*RMSE *= (*r*^*T*^*r/*(*N *- *p*))^1/2 ^    (20)

R2=[∑U^iUi−∑U^i∑U^i]2[∑U^2−(∑U^i)2][∑U2−(∑Ui)2]     (21)
 MathType@MTEF@5@5@+=feaafiart1ev1aaatCvAUfKttLearuWrP9MDH5MBPbIqV92AaeXatLxBI9gBaebbnrfifHhDYfgasaacH8akY=wiFfYdH8Gipec8Eeeu0xXdbba9frFj0=OqFfea0dXdd9vqai=hGuQ8kuc9pgc9s8qqaq=dirpe0xb9q8qiLsFr0=vr0=vr0dc8meaabaqaciaacaGaaeqabaqabeGadaaakeaacqWGsbGudaahaaWcbeqaaiabikdaYaaakiabg2da9maalaaabaGaei4waS1aaabqaeaacuWGvbqvgaqcamaaBaaaleaacqWGPbqAaeqaaaqabeqaniabggHiLdGccqWGvbqvdaWgaaWcbaGaemyAaKgabeaakiabgkHiTmaaqaeabaGafmyvauLbaKaadaWgaaWcbaGaemyAaKgabeaaaeqabeqdcqGHris5aOWaaabqaeaacuWGvbqvgaqcamaaBaaaleaacqWGPbqAaeqaaaqabeqaniabggHiLdGccqGGDbqxdaahaaWcbeqaaiabikdaYaaaaOqaaiabcUfaBnaaqaeabaGafmyvauLbaKaaaSqabeqaniabggHiLdGcdaahaaWcbeqaaiabikdaYaaakiabgkHiTiabcIcaOmaaqaeabaGafmyvauLbaKaadaWgaaWcbaGaemyAaKgabeaaaeqabeqdcqGHris5aOGaeiykaKYaaWbaaSqabeaacqaIYaGmaaGccqGGDbqxcqGGBbWwdaaeabqaaiabdwfavnaaCaaaleqabaGaeGOmaidaaOGaeyOeI0IaeiikaGYaaabqaeaacqWGvbqvdaWgaaWcbaGaemyAaKgabeaaaeqabeqdcqGHris5aOGaeiykaKYaaWbaaSqabeaacqaIYaGmaaaabeqab0GaeyyeIuoakiabc2faDbaacaWLjaGaaCzcaiabcIcaOiabikdaYiabigdaXiabcMcaPaaa@6A0A@

where *U*_*i *_and U^
 MathType@MTEF@5@5@+=feaafiart1ev1aaatCvAUfKttLearuWrP9MDH5MBPbIqV92AaeXatLxBI9gBaebbnrfifHhDYfgasaacH8akY=wiFfYdH8Gipec8Eeeu0xXdbba9frFj0=OqFfea0dXdd9vqai=hGuQ8kuc9pgc9s8qqaq=dirpe0xb9q8qiLsFr0=vr0=vr0dc8meaabaqaciaacaGaaeqabaqabeGadaaakeaacuWGvbqvgaqcaaaa@2DEF@_*i *_are the observed and predicted state variable (frap¯
 MathType@MTEF@5@5@+=feaafiart1ev1aaatCvAUfKttLearuWrP9MDH5MBPbIqV92AaeXatLxBI9gBaebbnrfifHhDYfgasaacH8akY=wiFfYdH8Gipec8Eeeu0xXdbba9frFj0=OqFfea0dXdd9vqai=hGuQ8kuc9pgc9s8qqaq=dirpe0xb9q8qiLsFr0=vr0=vr0dc8meaabaqaciaacaGaaeqabaqabeGadaaakeaadaqdaaqaaiabdAgaMjabdkhaYjabdggaHjabdchaWbaaaaa@3233@(*s*)), respectively.

## Results and discussion

### Scenario A: Simultaneous identification of transport and binding rate parameters

**Figure 3:**
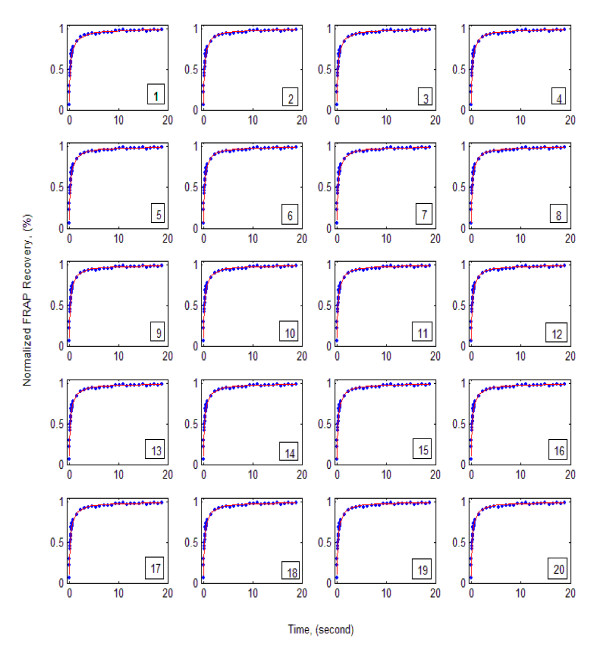
Predicted and experimental FRAP recovery curves for GFP-GR using one-site-mobile-immobile model (dots: Observed, solid lines: Simulated). Experimental data are from McNally (personal communication).

In this scenario, the aim was to estimate the transport and binding rate parameters for GFP-GR simultaneously by coupling the experimental data from the FRAP protocol, the Levenberg-Marquardt algorithm, and the numerical solution of Eq. (3). The results are given in Table 1 and Figure 3.

**Table 1:** The results of optimization for scenario A.

	Initial guesses			Optimized values								
run	*D*_*f *_(*μm*^2 ^*s*^-1^)	Ka* MathType@MTEF@5@5@+=feaafiart1ev1aaatCvAUfKttLearuWrP9MDH5MBPbIqV92AaeXatLxBI9gBaebbnrfifHhDYfgasaacH8akY=wiFfYdH8Gipec8Eeeu0xXdbba9frFj0=OqFfea0dXdd9vqai=hGuQ8kuc9pgc9s8qqaq=dirpe0xb9q8qiLsFr0=vr0=vr0dc8meaabaqaciaacaGaaeqabaqabeGadaaakeaacqWGlbWsdaqhaaWcbaGaemyyaegabaGaeiOkaOcaaaaa@301F@ (*s*^-1^)	*K*_*d *_(*s*^-1^)	*D*_*f *_(*μm*^2 ^*s*^-1^)	Ka* MathType@MTEF@5@5@+=feaafiart1ev1aaatCvAUfKttLearuWrP9MDH5MBPbIqV92AaeXatLxBI9gBaebbnrfifHhDYfgasaacH8akY=wiFfYdH8Gipec8Eeeu0xXdbba9frFj0=OqFfea0dXdd9vqai=hGuQ8kuc9pgc9s8qqaq=dirpe0xb9q8qiLsFr0=vr0=vr0dc8meaabaqaciaacaGaaeqabaqabeGadaaakeaacqWGlbWsdaqhaaWcbaGaemyyaegabaGaeiOkaOcaaaaa@301F@ (*s*^-1^)	*K*_*d *_(*s*^-1^)	*F*_*eq*_	*C*_*eq*_	*t*_*b *_(*ms*)	*t*_*d *_(*ms*)	*RMSE*	*R*^2^

1	1.4	0.01	0.24	1.3454	0.0081	0.249	0.9685	0.0315	4016	123450	0.0241	0.9904
2	15	500	86	13.5563	806	83	0.0934	0.9066	12.00	1.2407	0.0233	0.9912
3	10	20	50	1.2689	22.88	538	0.9592	0.0408	1.90	44.00	0.0245	0.9903
4	1.26	3000	5	79.7179	1.06*10^4^	168	0.0156	0.9844	6.00	9.00	0.0236	0.9910
5	12	30	490	1.8558	256	489	0.6564	0.3436	2.00	3.91	0.0244	0.9904
6	1.2	200	49	7.4289	200	42.5	0.1753	0.8247	23.50	5.00	0.0235	0.9911
7	7	2	470	1.2248	4.70	540.72	0.9914	0.0086	1.80	213.00	0.0245	0.993
8	0.7	202	0.047	6.6616	56.362	38.25	0.4043	0.5957	26.10	18.00	0.0235	0.9910
9	1.5	0.001	85	1.2127	7*10^-5^	91.21	1.000	0.000	11.00	15.00	0.0246	0.9902
10	1.5	0.1	1*10^-5^	1.2127	0.1874	1*10^-5^	0.0001	0.9999	200	5336	0.0245	0.9903
11	1.5	1*10^-5^	1	1.4652	0.1974	2.1902	0.9173	0.0827	456.6	5066	0.0251	0.9900
12	9.2	500	86.4	8.3315	468.56	83.38	0.1511	0.8489	12.00	2.00	0.0234	0.9911
13	25	0.001	100	1.2534	1.3557	44.94	0.9707	0.0293	22.30	738	0.0245	0.9903
14	0.25	0.001	100	1.2236	0.4235	119.71	0.9965	0.0035	8.40	2361	0.0245	0.9903
15	5	400	0.40	10.1911	396.8	56.7	0.1250	0.8750	17.60	2.52	0.0233	0.9911
16	15	4	1400	1.2205	3.81	1389	0.9973	0.0027	7.00	262	0.0245	0.9903
17	4.5	150	385	4.3970	986	380	0.2782	0.7218	2.60	1.00	0.0242	0.9905
18	10	150	385	8.861	2458	396	0.1388	0.8612	2.50	0.40	0.0242	0.9905
19	0.4	0.5	0.003	1.6371	0.5211	3.20	0.86	0.1400	312.50	1919	0.0254	0.9901
20^#^	-	-	-	9.20	500	86.4	0.1474	0.8526	11.60	2.00	0.0255	0.9886

# These values were obtained by Sprague *et al*. [14].

Analysis of Table 1 reveals several points regarding the mobility and binding of GFP-GR inside the nucleus. First, as pointed out in [14], the primary rate kinetics or single-binding state (Eq. 1) can satisfactorily describe the binding process of GFP-GR inside the nucleus. Therefore, we did not attempt to develop a two-site-mobile-immobile model to simulate the mobility and binding of GFP-GR.

Second, the values for mass transport and binding rate parameters estimated in [14] are given as run 20 in Table 1 and Figure 3 for sake of comparison. Table 1 and Figure 3 indicate many combinations of three parameters that give essentially the same error level (or objective function magnitude) and produce equally excellent fits (only 20 runs were reported). The values obtained in [14] represent only one of the possible solutions. In other words, the inverse problem is not well-posed and has no unique solution. This explains the conflicting parameter values that have been reported by investigators for a special biomolecule using the FRAP protocol. The reason for the ill-posedness of the inverse problem is that the FRAP protocol, though useful for studying the dynamics of cells, provides insufficient information to estimate mass transport and binding rate parameters of biomolecules uniquely and simultaneously.

Third, the optimized values of the free molecular diffusion coefficient for GFP-GR range from 1.2 to 79.7179 *μm*^2 ^*s*^-1^. Except for *D*_*f *_= 79.1719 *μm*^2^*s*^-1 ^the estimated values are physically reasonable. Note that we did not take into account the convective flux of GFP-GR toward the bleached area (in equations 2 and 3), which means that the optimized values of the molecular diffusion coefficient are somewhat overestimated in comparison to the "true" value.

Fourth, using Eqs. (6) and (7), Sprague et al. [14] concluded that 86% of the GFP-GR is bound and only 14% is free. Our study, however, indicates that using FRAP, one cannot say how much of the biomolecule is free and how much is bound. As Table 1 shows, the concentration of free GFP-GR ranges from zero to 100%. The same is true for the concentration of the bound complex. For instance, referring to the results obtained in run 9, one may conclude that 100% of the GFP-GR is free, while the results of run 10 show that all of it is bound. Note that both these runs produce excellent fits with the same *RMSE *and coefficient of determination (see Figure 3: scenarios 9 and 10).

Fifth, the average binding time per vacant site, calculated by *t*_*b *_= 1/*K*_*d *_[14], varies between 0.72 ms and 4.016 s. Again this shows that the findings of [14], that the average binding time per vacant site for GFP-GR is 12.7 ms, represent just one the possible values. Similarly, the average time for diffusion of GFP-GR from one binding site to the next, obtained by *t*_*d *_= 1/Ka*
 MathType@MTEF@5@5@+=feaafiart1ev1aaatCvAUfKttLearuWrP9MDH5MBPbIqV92AaeXatLxBI9gBaebbnrfifHhDYfgasaacH8akY=wiFfYdH8Gipec8Eeeu0xXdbba9frFj0=OqFfea0dXdd9vqai=hGuQ8kuc9pgc9s8qqaq=dirpe0xb9q8qiLsFr0=vr0=vr0dc8meaabaqaciaacaGaaeqabaqabeGadaaakeaacqWGlbWsdaqhaaWcbaGaemyyaegabaGaeiOkaOcaaaaa@301F@[42], ranges between 0.4 ms to 34.3 hours (1.2345*10^5 ^s). The broad range of *t*_*d *_for GFP-GR indicates that it is meaningless to give an average time for macro-molecule diffusion inside living cells.

Overall, these results indicate that using experimental data from the FRAP protocol and coupling it with curve fitting methods, one cannot draw conclusions regarding binding reaction, slow or rapid mobility of biomolecules, and concentrations of free macromolecule, vacant binding sites and bound complex inside living cells. The results of parameter estimation should be coupled with other experimental studies and large scale optimization techniques such as Monte-Carlo simulation to prevent misleading conclusions and inferences.

### Scenario B: Estimation of a single parameter in a FRAP experiment

In this scenario, two of the three parameters were kept at their "true" values and the optimized value of the third parameter was estimated. The optimization algorithm was used to estimate a single parameter for both noise-free and noisy data and the results are presented in Tables 2, 3, 4. The values inside parentheses are for noisy data. As these tables show, the FRAP protocol provides enough information to estimate one parameter uniquely if the other two are known. This is true for both noise-free and noisy data. The other important finding is the robustness and efficiency of the developed optimization algorithm, which converged to the "true" values of the parameters regardless of the initial guesses (compare the initial guesses for the parameters with the optimized values).

**Table 2:** The results of parameter optimization for scenario B (estimation of molecular diffusion coefficient in a FRAP experiment).

Estimate *D*_*f*_							
Initial guesses			Optimized values				

*D*_*f *_(*μm*^2 ^*s*^-1^)	Ka* MathType@MTEF@5@5@+=feaafiart1ev1aaatCvAUfKttLearuWrP9MDH5MBPbIqV92AaeXatLxBI9gBaebbnrfifHhDYfgasaacH8akY=wiFfYdH8Gipec8Eeeu0xXdbba9frFj0=OqFfea0dXdd9vqai=hGuQ8kuc9pgc9s8qqaq=dirpe0xb9q8qiLsFr0=vr0=vr0dc8meaabaqaciaacaGaaeqabaqabeGadaaakeaacqWGlbWsdaqhaaWcbaGaemyyaegabaGaeiOkaOcaaaaa@301F@ (*s*^-1^)	*K*_*d *_(*s*^-1^)	*D*_*f *_(*μm*^2 ^*s*^-1^)	Ka* MathType@MTEF@5@5@+=feaafiart1ev1aaatCvAUfKttLearuWrP9MDH5MBPbIqV92AaeXatLxBI9gBaebbnrfifHhDYfgasaacH8akY=wiFfYdH8Gipec8Eeeu0xXdbba9frFj0=OqFfea0dXdd9vqai=hGuQ8kuc9pgc9s8qqaq=dirpe0xb9q8qiLsFr0=vr0=vr0dc8meaabaqaciaacaGaaeqabaqabeGadaaakeaacqWGlbWsdaqhaaWcbaGaemyyaegabaGaeiOkaOcaaaaa@301F@ (*s*^-1^)	*K*_*d *_(*s*^-1^)	*RMSE*	*R*^2^

3	30	0.1108	29.9975 (29.8032)	30	0.1108	0.00 (0.01)	1.0000 (0.9984)
5	30	0.1108	29.9968 (29.7362)	30	0.1108	0.00 (0.01)	1.0000 (0.9984)
10	30	0.1108	29.9968 (29.7978)	30	0.1108	0.00 (0.01)	1.0000 (0.9984)
15	30	0.1108	29.9959 (29.7483)	30	0.1108	0.00 (0.01)	1.0000 (0.9984)
20	30	0.1108	29.9972 (29.7490)	30	0.1108	0.00 (0.01)	1.0000 (0.9984)
45	30	0.1108	29.9974 (29.7376)	30	0.1108	0.00 (0.01)	1.0000 (0.9984)
1000	30	0.1108	29.9973 (29.7507)	30	0.1108	0.00 (0.01)	1.0000 (0.9984)
500	30	0.1108	29.9969 (29.7910)	30	0.1108	0.00 (0.01)	1.0000 (0.9984)

The values in parentheses were obtained using corrupted data.

**Table 3:** The results of parameter optimization for scenario B (estimation of pseudo-association rate coefficient in a FRAP experiment).

Estimate Ka* MathType@MTEF@5@5@+=feaafiart1ev1aaatCvAUfKttLearuWrP9MDH5MBPbIqV92AaeXatLxBI9gBaebbnrfifHhDYfgasaacH8akY=wiFfYdH8Gipec8Eeeu0xXdbba9frFj0=OqFfea0dXdd9vqai=hGuQ8kuc9pgc9s8qqaq=dirpe0xb9q8qiLsFr0=vr0=vr0dc8meaabaqaciaacaGaaeqabaqabeGadaaakeaacqWGlbWsdaqhaaWcbaGaemyyaegabaGaeiOkaOcaaaaa@301F@							
Initial guesses			Optimized values				

*D*_*f *_(*μm*^2 ^*s*^-1^)	Ka* MathType@MTEF@5@5@+=feaafiart1ev1aaatCvAUfKttLearuWrP9MDH5MBPbIqV92AaeXatLxBI9gBaebbnrfifHhDYfgasaacH8akY=wiFfYdH8Gipec8Eeeu0xXdbba9frFj0=OqFfea0dXdd9vqai=hGuQ8kuc9pgc9s8qqaq=dirpe0xb9q8qiLsFr0=vr0=vr0dc8meaabaqaciaacaGaaeqabaqabeGadaaakeaacqWGlbWsdaqhaaWcbaGaemyyaegabaGaeiOkaOcaaaaa@301F@ (*s*^-1^)	*K*_*d *_(*s*^-1^)	*D*_*f *_(*μm*^2 ^*s*^-1^)	Ka* MathType@MTEF@5@5@+=feaafiart1ev1aaatCvAUfKttLearuWrP9MDH5MBPbIqV92AaeXatLxBI9gBaebbnrfifHhDYfgasaacH8akY=wiFfYdH8Gipec8Eeeu0xXdbba9frFj0=OqFfea0dXdd9vqai=hGuQ8kuc9pgc9s8qqaq=dirpe0xb9q8qiLsFr0=vr0=vr0dc8meaabaqaciaacaGaaeqabaqabeGadaaakeaacqWGlbWsdaqhaaWcbaGaemyyaegabaGaeiOkaOcaaaaa@301F@ (*s*^-1^)	*K*_*d *_(*s*^-1^)	*RMSE*	*R*^2^

30	3.00	0.1108	30	30.0032 (30.3523)	0.1108	0.00 (0.01)	1.000 (0.998)
30	1 × 10^-3^	0.1108	30	29.9982 (30.2455)	0.1108	0.00 (0.01)	1.000 (0.998)
30	1 × 10^-6^	0.1108	30	30.0031 (30.2468)	0.1108	0.00 (0.01)	1.000 (0.998)
30	1 × 10^6^	0.1108	30	30.0030 (30.2478)	0.1108	0.00 (0.01)	1.000 (0.998)
30	1 × 10^3^	0.1108	30	30.0031 (30.2507)	0.1108	0.00 (0.01)	1.000 (0.998)
30	300.00	0.1108	30	30.0030 (30.3188)	0.1108	0.00 (0.01)	1.000 (0.998)
30	10.00	0.1108	30	30.0030 (30.2115)	0.1108	0.00 (0.01)	1.000 (0.998)
30	0.050	0.1108	30	30.0030 (30.1655)	0.1108	0.00 (0.01)	1.000 (0.998)

The values in parentheses were obtained using corrupted data.

**Table 4:** The results of parameter optimization for scenario B (estimation of dissociation rate coefficient in a FRAP experiment).

Estimate *K*_*d*_							
Initial guesses			Optimized values				

*D*_*f *_(*μm*^2 ^*s*^-1^)	Ka* MathType@MTEF@5@5@+=feaafiart1ev1aaatCvAUfKttLearuWrP9MDH5MBPbIqV92AaeXatLxBI9gBaebbnrfifHhDYfgasaacH8akY=wiFfYdH8Gipec8Eeeu0xXdbba9frFj0=OqFfea0dXdd9vqai=hGuQ8kuc9pgc9s8qqaq=dirpe0xb9q8qiLsFr0=vr0=vr0dc8meaabaqaciaacaGaaeqabaqabeGadaaakeaacqWGlbWsdaqhaaWcbaGaemyyaegabaGaeiOkaOcaaaaa@301F@ (*s*^-1^)	*K*_*d *_(*s*^-1^)	*D*_*f *_(*μm*^2 ^*s*^-1^)	Ka* MathType@MTEF@5@5@+=feaafiart1ev1aaatCvAUfKttLearuWrP9MDH5MBPbIqV92AaeXatLxBI9gBaebbnrfifHhDYfgasaacH8akY=wiFfYdH8Gipec8Eeeu0xXdbba9frFj0=OqFfea0dXdd9vqai=hGuQ8kuc9pgc9s8qqaq=dirpe0xb9q8qiLsFr0=vr0=vr0dc8meaabaqaciaacaGaaeqabaqabeGadaaakeaacqWGlbWsdaqhaaWcbaGaemyyaegabaGaeiOkaOcaaaaa@301F@ (*s*^-1^)	*K*_*d *_(*s*^-1^)	*RMSE*	*R*^2^

30	30	0.0008	30	30	0.1108 (0.1107)	0.0000 (0.0102)	1.000 (0.998)
30	300	0.8000	30	30	0.1108 (0.1107)	0.0000 (0.0102)	1.000 (0.998)
30	30	0.0001	30	30	0.1108 (0.1107)	0.0000 (0.0102)	1.000 (0.998)
30	30	1.0000	30	30	0.1108 (0.1107)	0.0000 (0.0102)	1.000 (0.998)
30	30	0.0500	30	30	0.1108 (0.1107)	0.0000 (0.0102)	1.000 (0.998)
30	30	0.0010	30	30	0.1108 (0.1107)	0.0000 (0.0102)	1.000 (0.998)
30	30	1 × 10^-5^	30	30	0.1108 (0.1108)	0.0000 (0.0102)	1.000 (0.998)
30	30	1 × 10^-6^	30	30	0.1108 (0.1107)	0.0000 (0.0102)	1.000 (0.998)

The values in parentheses were obtained using corrupted data.

### Scenario C: Estimation of two parameters in a FRAP experiment

In this scenario, the optimized values of the binding rate coefficients were first estimated given that the "true" value of the molecular diffusion coefficient of GFP-GR was known. Again, the optimization algorithm was used for both noise-free and noisy data and the results are given in Table 5. As Table 5 indicates, using the FRAP experiment coupled with the proposed inverse modeling strategy, one can estimate the individual values (not just the ratio) of the binding rate coefficients uniquely if the value of the diffusion coefficient is known. This is true for both noise-free and noisy data.

**Table 5:** The results of parameter optimization for scenario C (estimation of two parameters in a FRAP experiment: Ka*
 MathType@MTEF@5@5@+=feaafiart1ev1aaatCvAUfKttLearuWrP9MDH5MBPbIqV92AaeXatLxBI9gBaebbnrfifHhDYfgasaacH8akY=wiFfYdH8Gipec8Eeeu0xXdbba9frFj0=OqFfea0dXdd9vqai=hGuQ8kuc9pgc9s8qqaq=dirpe0xb9q8qiLsFr0=vr0=vr0dc8meaabaqaciaacaGaaeqabaqabeGadaaakeaacqWGlbWsdaqhaaWcbaGaemyyaegabaGaeiOkaOcaaaaa@301F@ - *K*_*d*_).

Estimate *K*_*a *_and *K*_*d*_							
Initial guesses			Optimized values				

*D*_*f *_(*μm*^2 ^*s*^-1^)	Ka* MathType@MTEF@5@5@+=feaafiart1ev1aaatCvAUfKttLearuWrP9MDH5MBPbIqV92AaeXatLxBI9gBaebbnrfifHhDYfgasaacH8akY=wiFfYdH8Gipec8Eeeu0xXdbba9frFj0=OqFfea0dXdd9vqai=hGuQ8kuc9pgc9s8qqaq=dirpe0xb9q8qiLsFr0=vr0=vr0dc8meaabaqaciaacaGaaeqabaqabeGadaaakeaacqWGlbWsdaqhaaWcbaGaemyyaegabaGaeiOkaOcaaaaa@301F@ (*s*^-1^)	*K*_*d *_(*s*^-1^)	*D*_*f *_(*μm*^2^*s*^-1^)	Ka* MathType@MTEF@5@5@+=feaafiart1ev1aaatCvAUfKttLearuWrP9MDH5MBPbIqV92AaeXatLxBI9gBaebbnrfifHhDYfgasaacH8akY=wiFfYdH8Gipec8Eeeu0xXdbba9frFj0=OqFfea0dXdd9vqai=hGuQ8kuc9pgc9s8qqaq=dirpe0xb9q8qiLsFr0=vr0=vr0dc8meaabaqaciaacaGaaeqabaqabeGadaaakeaacqWGlbWsdaqhaaWcbaGaemyyaegabaGaeiOkaOcaaaaa@301F@ (*s*^-1^)	*K*_*d *_(*s*^-1^)	*RMSE*	*R*^2^

30	90	0.005	30	30.0246 (32.7366)	0.1108 (0.1122)	0.00 (0.01)	1.000 (0.99)
30	20	0.01	30	29.9762 (28.8955)	0.1108 (0.1101)	0.00 (0.01)	1.000 (0.99)
30	250	0.01	30	30.0729 (33.7009)	0.1108 (0.1128)	0.00 (0.01)	1.000 (0.99)
30	435	0.0005	30	30.1108 (34.2443)	0.1108 (0.1131)	0.00 (0.01)	1.000 (0.99)
30	10	0.01	30	29.9576 (31.8386)	0.1108 (0.1118)	0.00 (0.01)	1.000 (0.99)
30	100	1	30	30.0027 (36.0428)	0.1108 (0.1141)	0.00 (0.01)	1.000 (0.99)
30	100	2*10^6^	30	30.0209 (33.8034)	0.1108 (0.1129)	0.00 (0.01)	1.000 (0.99)
30	1000	0.5	30	30.0082 (32.7609)	0.1108 (0.1122)	0.00 (0.01)	1.000 (0.99)

The values in parentheses were obtained using corrupted data.

We then tried to identify the optimized values of the molecular diffusion coefficient and dissociation rate coefficient for both noise-free and noisy data given that Ka*
 MathType@MTEF@5@5@+=feaafiart1ev1aaatCvAUfKttLearuWrP9MDH5MBPbIqV92AaeXatLxBI9gBaebbnrfifHhDYfgasaacH8akY=wiFfYdH8Gipec8Eeeu0xXdbba9frFj0=OqFfea0dXdd9vqai=hGuQ8kuc9pgc9s8qqaq=dirpe0xb9q8qiLsFr0=vr0=vr0dc8meaabaqaciaacaGaaeqabaqabeGadaaakeaacqWGlbWsdaqhaaWcbaGaemyyaegabaGaeiOkaOcaaaaa@301F@ is known. The results are presented in Table 6, which indicates that the FRAP protocol provides enough information to estimate the molecular diffusion coefficient and dissociation rate parameter uniquely for both noise-free and noisy data.

**Table 6:** The results of parameter optimization for scenario C (estimation of two parameters in a FRAP experiment: *D*_*f *_- *K*_*d*_).

Estimate *D*_*f *_and *K*_*d*_							
Initial guesses			Optimized values				

*D*_*f *_(*μm*^2 ^*s*^-1^)	Ka* MathType@MTEF@5@5@+=feaafiart1ev1aaatCvAUfKttLearuWrP9MDH5MBPbIqV92AaeXatLxBI9gBaebbnrfifHhDYfgasaacH8akY=wiFfYdH8Gipec8Eeeu0xXdbba9frFj0=OqFfea0dXdd9vqai=hGuQ8kuc9pgc9s8qqaq=dirpe0xb9q8qiLsFr0=vr0=vr0dc8meaabaqaciaacaGaaeqabaqabeGadaaakeaacqWGlbWsdaqhaaWcbaGaemyyaegabaGaeiOkaOcaaaaa@301F@ (*s*^-1^)	*K*_*d *_(*s*^-1^)	*D*_*f *_(*μm*^2 ^*s*^-1^)	Ka* MathType@MTEF@5@5@+=feaafiart1ev1aaatCvAUfKttLearuWrP9MDH5MBPbIqV92AaeXatLxBI9gBaebbnrfifHhDYfgasaacH8akY=wiFfYdH8Gipec8Eeeu0xXdbba9frFj0=OqFfea0dXdd9vqai=hGuQ8kuc9pgc9s8qqaq=dirpe0xb9q8qiLsFr0=vr0=vr0dc8meaabaqaciaacaGaaeqabaqabeGadaaakeaacqWGlbWsdaqhaaWcbaGaemyyaegabaGaeiOkaOcaaaaa@301F@ (*s*^-1^)	*K*_*d *_(*s*^-1^)	*RMSE*	*R*^2^

8	30	0.008	30.0111 (27.528)	30	0.1108 (0.1122)	0.000 (0.0101)	1.000 (0.998)
48	30	0.08	29.9972 (29.935)	30	0.1108 (0.1108)	0.000 (0.0101)	1.000 (0.998)
8	30	1	29.9989 (28.204)	30	0.1108 (0.1118)	0.000 (0.0101)	1.000 (0.998)
80	30	1	30.0100 (28.294)	30	0.1108 (0.1117)	0.000 (0.0101)	1.000 (0.998)
150	30	0.01	30.0156 (36.477)	30	0.1108 (0.1077)	0.000 (0.0104)	1.000 (0.998)
0.150	30	0.1	30.0005 (27.822)	30	0.1108 (0.1120)	0.000 (0.0101)	1.000 (0.998)
15	30	0.001	30.0090 (24.555)	30	0.1108 (0.1143)	0.000 (0.0102)	1.000 (0.998)
150	30	0.001	30.0142 (188.225)	30	0.1108 (0.0946)	0.000 (0.0133)	1.000 (0.998)

The values in parentheses were obtained using corrupted data.

Finally, we tried to estimate the optimized values of the free molecular diffusion coefficient and pseudo-association rate parameter by fixing *K*_*d *_at the "true" value for both noise-free and noisy data. The results are shown in Table 7. This table indicates that the FRAP experiment provides insufficient information for unique simultaneous estimation of the molecular diffusion coefficient and the pseudo-association rate parameter even for noise-free data. One must know one of them and try to estimate the other from the FRAP data using the inverse modeling strategy.

**Table 7:** The results of parameter optimization for scenario C (estimation of two parameters in a FRAP experiment: *D*_*f *_- Ka*
 MathType@MTEF@5@5@+=feaafiart1ev1aaatCvAUfKttLearuWrP9MDH5MBPbIqV92AaeXatLxBI9gBaebbnrfifHhDYfgasaacH8akY=wiFfYdH8Gipec8Eeeu0xXdbba9frFj0=OqFfea0dXdd9vqai=hGuQ8kuc9pgc9s8qqaq=dirpe0xb9q8qiLsFr0=vr0=vr0dc8meaabaqaciaacaGaaeqabaqabeGadaaakeaacqWGlbWsdaqhaaWcbaGaemyyaegabaGaeiOkaOcaaaaa@301F@).

Estimate *D*_*f *_and Ka* MathType@MTEF@5@5@+=feaafiart1ev1aaatCvAUfKttLearuWrP9MDH5MBPbIqV92AaeXatLxBI9gBaebbnrfifHhDYfgasaacH8akY=wiFfYdH8Gipec8Eeeu0xXdbba9frFj0=OqFfea0dXdd9vqai=hGuQ8kuc9pgc9s8qqaq=dirpe0xb9q8qiLsFr0=vr0=vr0dc8meaabaqaciaacaGaaeqabaqabeGadaaakeaacqWGlbWsdaqhaaWcbaGaemyyaegabaGaeiOkaOcaaaaa@301F@							
Initial guesses			Optimized values				

*D*_*f *_(*μm*^2 ^*s*^-1^)	Ka* MathType@MTEF@5@5@+=feaafiart1ev1aaatCvAUfKttLearuWrP9MDH5MBPbIqV92AaeXatLxBI9gBaebbnrfifHhDYfgasaacH8akY=wiFfYdH8Gipec8Eeeu0xXdbba9frFj0=OqFfea0dXdd9vqai=hGuQ8kuc9pgc9s8qqaq=dirpe0xb9q8qiLsFr0=vr0=vr0dc8meaabaqaciaacaGaaeqabaqabeGadaaakeaacqWGlbWsdaqhaaWcbaGaemyyaegabaGaeiOkaOcaaaaa@301F@ (*s*^-1^)	*K*_*d *_(*s*^-1^)	*D*_*f *_(*μm*^2 ^*s*^-1^)	Ka* MathType@MTEF@5@5@+=feaafiart1ev1aaatCvAUfKttLearuWrP9MDH5MBPbIqV92AaeXatLxBI9gBaebbnrfifHhDYfgasaacH8akY=wiFfYdH8Gipec8Eeeu0xXdbba9frFj0=OqFfea0dXdd9vqai=hGuQ8kuc9pgc9s8qqaq=dirpe0xb9q8qiLsFr0=vr0=vr0dc8meaabaqaciaacaGaaeqabaqabeGadaaakeaacqWGlbWsdaqhaaWcbaGaemyyaegabaGaeiOkaOcaaaaa@301F@ (*s*^-1^)	*K*_*d *_(*s*^-1^)	*RMSE*	*R*^2^

50	3	0.1108	3.8162 (6.9081)	4.1352 (7.2953)	0.1108	0.0042 (0.0104)	0.9997 (0.9983)
3	50	0.1108	47.7952 (35.6424)	47.5709 (35.8541)	0.1108	0.0003 (0.0102)	1.0000 (0.9984)
20	25	0.1108	25.2109 (25.2109)	25.2769 (25.2769)	0.1108	0.0001 (0.0001)	1.0000 (1.0000)
25	20	0.1108	24.5737 (24.5737)	24.6488 (24.6488)	0.1108	0.0002 (0.0002)	1.0000 (1.0000)
28	35	0.1108	34.8874 (34.8874)	34.8255 (34.8255)	0.1108	0.0001 (0.0001)	1.0000 (1.0000)
0.1	100	0.1108	89.9205 (89.9205)	89.1097 (89.1097)	0.1108	0.0005 (0.0005)	1.0000 (1.0000)
100	0.1	0.1108	8.5511 (8.5511)	8.8336 (8.8336)	0.1108	0.0017 (0.0017)	1.0000 (1.0000)
28	28	0.1108	28.2015 (28.2015)	28.2282 (28.2282)	0.1108	0.0001 (0.0001)	1.0000 (1.0000)

The values in parentheses were obtained using corrupted data.

It can be argued that the reason for the non-uniqueness of the inverse problem lies in the relationship between the free molecular diffusion coefficient and the pseudo-association rate parameter. To investigate the possibility of high intercorrelation between these two parameters further, the parameter covariance matrix was calculated [37]:

C=se2(JTJ)−1     (22)
 MathType@MTEF@5@5@+=feaafiart1ev1aaatCvAUfKttLearuWrP9MDH5MBPbIqV92AaeXatLxBI9gBaebbnrfifHhDYfgasaacH8akY=wiFfYdH8Gipec8Eeeu0xXdbba9frFj0=OqFfea0dXdd9vqai=hGuQ8kuc9pgc9s8qqaq=dirpe0xb9q8qiLsFr0=vr0=vr0dc8meaabaqaciaacaGaaeqabaqabeGadaaakeaacqWGdbWqcqGH9aqpcqWGZbWCdaqhaaWcbaGaemyzaugabaGaeGOmaidaaOWaaeWaaeaacqWGkbGsdaahaaWcbeqaaiabdsfaubaakiabdQeakbGaayjkaiaawMcaamaaCaaaleqabaGaeyOeI0IaeGymaedaaOGaaCzcaiaaxMaacqGGOaakcqaIYaGmcqaIYaGmcqGGPaqkaaa@3EC5@

where *C *is the first-order approximation of the parameter covariance matrix, *J *is the final optimized Jacobian matrix, which can easily be obtained at the end of optimization, and *s*_*e *_is the estimated error variance [27]:

*s*_*e *_= *r*^*T *^*r*/(*N *- *p*)     (23)

The diagonal elements of the parameter covariance matrix are variances and the off-diagonal elements are the covariances between the parameters. Using this matrix, one can calculate the parameter correlation matrix (also known as the variance-covariance matrix), which is a square matrix [27]:

*COR *(*P*)_*ij *_= *C*_*ij*_/[(*C*_*ii*_)^1/2 ^(*C*_*jj*_)^1/2^]    (24)

Equation (24) identifies the degree of linear correlation between the optimized parameters. In other words, the correlation matrix quantifies the nonorthogonality between two parameters. A value of ± 1 reflects perfect linear correlation between two parameters whereas 0 indicates no correlation at all. The matrix may be used to identify which parameter, if any, is kept constant in the parameter optimization process because of high intercorrelation [41]. The correlation matrix for scenario C was found to be:

COR(P)=[1.00000.9890−0.24870.98901.0000−0.1196−0.2487−0.1196   1.0000]
 MathType@MTEF@5@5@+=feaafiart1ev1aaatCvAUfKttLearuWrP9MDH5MBPbIqV92AaeXatLxBI9gBaebbnrfifHhDYfgasaacH8akY=wiFfYdH8Gipec8Eeeu0xXdbba9frFj0=OqFfea0dXdd9vqai=hGuQ8kuc9pgc9s8qqaq=dirpe0xb9q8qiLsFr0=vr0=vr0dc8meaabaqaciaacaGaaeqabaqabeGadaaakeaacqWGdbWqcqWGpbWtcqWGsbGudaqadaqaaiabdcfaqbGaayjkaiaawMcaaiabg2da9maadmaabaqbaeaabmWaaaqaaiabigdaXiabc6caUiabicdaWiabicdaWiabicdaWiabicdaWaqaaiabicdaWiabc6caUiabiMda5iabiIda4iabiMda5iabicdaWaqaaiabgkHiTiabicdaWiabc6caUiabikdaYiabisda0iabiIda4iabiEda3aqaaiabicdaWiabc6caUiabiMda5iabiIda4iabiMda5iabicdaWaqaaiabigdaXiabc6caUiabicdaWiabicdaWiabicdaWiabicdaWaqaaiabgkHiTiabicdaWiabc6caUiabigdaXiabigdaXiabiMda5iabiAda2aqaaiabgkHiTiabicdaWiabc6caUiabikdaYiabisda0iabiIda4iabiEda3aqaaiabgkHiTiabicdaWiabc6caUiabigdaXiabigdaXiabiMda5iabiAda2aqaaiaaykW7caaMc8UaaGjbVlabigdaXiabc6caUiabicdaWiabicdaWiabicdaWiabicdaWaaaaiaawUfacaGLDbaaaaa@7106@

where the diagonal elements of the matrix are the correlations of each parameter with itself (i.e. unity).

The correlation between the molecular diffusion coefficient and the pseudo-association rate parameter is rDf−Ka*
 MathType@MTEF@5@5@+=feaafiart1ev1aaatCvAUfKttLearuWrP9MDH5MBPbIqV92AaeXatLxBI9gBaebbnrfifHhDYfgasaacH8akY=wiFfYdH8Gipec8Eeeu0xXdbba9frFj0=OqFfea0dXdd9vqai=hGuQ8kuc9pgc9s8qqaq=dirpe0xb9q8qiLsFr0=vr0=vr0dc8meaabaqaciaacaGaaeqabaqabeGadaaakeaacqWGYbGCdaWgaaWcbaGaemiraq0aaSbaaWqaaiabdAgaMbqabaWccqGHsislcqWGlbWsdaqhaaadbaGaemyyaegabaGaeiOkaOcaaaWcbeaaaaa@354F@ = 0.989, and those between the molecular diffusion coefficient-dissociation rate parameter and reaction rate coefficients are rDf−Kd
 MathType@MTEF@5@5@+=feaafiart1ev1aaatCvAUfKttLearuWrP9MDH5MBPbIqV92AaeXatLxBI9gBaebbnrfifHhDYfgasaacH8akY=wiFfYdH8Gipec8Eeeu0xXdbba9frFj0=OqFfea0dXdd9vqai=hGuQ8kuc9pgc9s8qqaq=dirpe0xb9q8qiLsFr0=vr0=vr0dc8meaabaqaciaacaGaaeqabaqabeGadaaakeaacqWGYbGCdaWgaaWcbaGaemiraq0aaSbaaWqaaiabdAgaMbqabaWccqGHsislcqWGlbWsdaWgaaadbaGaemizaqgabeaaaSqabaaaaa@3478@ = -0.2487 and rKa*−Kd
 MathType@MTEF@5@5@+=feaafiart1ev1aaatCvAUfKttLearuWrP9MDH5MBPbIqV92AaeXatLxBI9gBaebbnrfifHhDYfgasaacH8akY=wiFfYdH8Gipec8Eeeu0xXdbba9frFj0=OqFfea0dXdd9vqai=hGuQ8kuc9pgc9s8qqaq=dirpe0xb9q8qiLsFr0=vr0=vr0dc8meaabaqaciaacaGaaeqabaqabeGadaaakeaacqWGYbGCdaWgaaWcbaGaem4saS0aa0baaWqaaiabdggaHbqaaiabcQcaQaaaliabgkHiTiabdUealnaaBaaameaacqWGKbazaeqaaaWcbeaaaaa@3559@ = -0.1196, respectively. The signs of the elements of the correlation matrix are physically reasonable because on the basis of the primary rate kinetics, Eq. (1), we expect a negative correlation between *D*_*f *_and *K*_*d *_as well as between *K*_*a *_and *K*_*d*_. We also expect a positive correlation between *K*_*a *_and *D*_*f*_.

The high intercorrelation between the molecular diffusion coefficient and the pseudo-association rate coefficient makes it impossible to obtain a unique solution for the inverse problem using experimental data from the FRAP protocol. The common practice in these situations is to fix one parameter and estimate the other by parameter optimization algorithms.

### Scenario D: Estimation of three parameters for noise-free FRAP data

In this scenario we tried to estimate the optimized values of the mass transport and binding rate coefficients for noise-free data. As Table 8 indicates, the FRAP experiment provides insufficient information for unique simultaneous estimation of the mass transport and binding rate parameters even for noise-free data. The reason, as discussed above, is the high intercorrelation between the molecular diffusion coefficient and the pseudo-association rate parameter.

**Table 8:** The results of parameter optimization for scenario D (estimation of three parameters for noise-free FRAP data).

Estimate *D*_*f*_, *K*_*d*_, and Ka* MathType@MTEF@5@5@+=feaafiart1ev1aaatCvAUfKttLearuWrP9MDH5MBPbIqV92AaeXatLxBI9gBaebbnrfifHhDYfgasaacH8akY=wiFfYdH8Gipec8Eeeu0xXdbba9frFj0=OqFfea0dXdd9vqai=hGuQ8kuc9pgc9s8qqaq=dirpe0xb9q8qiLsFr0=vr0=vr0dc8meaabaqaciaacaGaaeqabaqabeGadaaakeaacqWGlbWsdaqhaaWcbaGaemyyaegabaGaeiOkaOcaaaaa@301F@							
Initial guesses			Optimized values				

*D*_*f *_(*μm*^2 ^*s*^-1^)	Ka* MathType@MTEF@5@5@+=feaafiart1ev1aaatCvAUfKttLearuWrP9MDH5MBPbIqV92AaeXatLxBI9gBaebbnrfifHhDYfgasaacH8akY=wiFfYdH8Gipec8Eeeu0xXdbba9frFj0=OqFfea0dXdd9vqai=hGuQ8kuc9pgc9s8qqaq=dirpe0xb9q8qiLsFr0=vr0=vr0dc8meaabaqaciaacaGaaeqabaqabeGadaaakeaacqWGlbWsdaqhaaWcbaGaemyyaegabaGaeiOkaOcaaaaa@301F@ (*s*^-1^)	*K*_*d *_(*s*^-1^)	*D*_*f *_(*μm*^2 ^*s*^-1^)	Ka* MathType@MTEF@5@5@+=feaafiart1ev1aaatCvAUfKttLearuWrP9MDH5MBPbIqV92AaeXatLxBI9gBaebbnrfifHhDYfgasaacH8akY=wiFfYdH8Gipec8Eeeu0xXdbba9frFj0=OqFfea0dXdd9vqai=hGuQ8kuc9pgc9s8qqaq=dirpe0xb9q8qiLsFr0=vr0=vr0dc8meaabaqaciaacaGaaeqabaqabeGadaaakeaacqWGlbWsdaqhaaWcbaGaemyyaegabaGaeiOkaOcaaaaa@301F@ (*s*^-1^)	*K*_*d *_(*s*^-1^)	*RMSE*	*R*^2^

20	43	0.01	41.8564	42.7664	0.1112	0.0002	1.0000
200	43	0.01	170.9403	166.9715	0.1106	0.0006	1.0000
27	28	0.01	27.7434	27.6444	0.1107	0.0001	1.0000
29	29	0.01	29.0008	29.0018	0.1108	0.0000	1.0000
29	29	0.001	21.8680	21.5410	0.1104	0.0002	1.0000
29	290	0.0001	276.5849	287.3558	0.1117	0.0005	1.0000
15	500	0.0001	462.2080	491.3985	0.1121	0.0005	1.0000
15	0.5	0.8	3.65890	3.6106	0.1087	0.0043	0.9997

The values in parentheses were obtained using corrupted data.

## Unique parameter identification

The optimization scenarios considered above show a possible way of obtaining unique values for diffusion coefficient and binding rate parameters of biomolecules inside living cells. A possible procedure for obtaining unique values for molecular diffusion coefficient and reaction rate parameters of macro-molecules is to conduct two FRAP experiments in different regimes on the same class of cell and biomolecule. One experiment should be conducted in an effective diffusion regime to estimate diffusion coefficient independent of binding. The other should be performed in reaction dominant or diffusion-reaction dominant regimes to identify the binding rate parameters. Conducting two FRAP experiments in two different regimes is, however, beyond the scope of the present study. It will be pursued in future research.

## Posedness analysis of the inverse problem

To study the non-uniqueness problem from another angle, we performed a posedness analysis of the inverse problem. A problem is ill-posed when it either has no solution at all, or no unique solution, or the solution is not stable [43]. Generally, ill-posedness in an inverse problem arises from non-uniqueness and instability. To investigate the ill-posedness of the inverse problem, we analyzed both its stability and its uniqueness.

### Stability analysis

Instability occurs when the estimated parameters are excessively sensitive to the input data. Any small errors in measurements will then lead to significant error in estimated values of parameters [27]. To perform the stability analysis, the data sets were corrupted by adding *N*(0, *σ*^2^) noise to each measurement. The resulting noisy data were then used as input for parameter optimization algorithm. The results are given in Tables 2 to 7 in parentheses. As these tables show, small changes in the input data generate no significant changes in the optimized values of the parameters. Therefore, the cause of the ill-posedness of the inverse problem is not instability.

### Uniqueness analysis

Non-uniqueness occurs when multiple parameter vectors can produce almost the same values of the objective function, thus making it impossible to obtain a unique solution [27]. This problem is closely related to parameter identifiability. In other words, is it possible to obtain accurate values for parameters in the mathematical model from the available experimental data? Parameter identifiability depends on both the structure of the mathematical model and the experimental data used. A common cause for non-identifiability of model parameters is, as noted in previous section, high intercorrelation among parameters. In these situations a change in one parameter generates a corresponding change in the correlated parameter making it impossible to obtain accurate estimates of either. Furthermore, even when parameters in a mathematical model are independent of each other, the experimental data may produce an objective function that is not sensitive enough to one or more parameters. The characteristic of the second situation is wide confidence regions on the estimated parameters and large estimation variances.

Whereas the only solution for the first case is to fix one of the parameters and estimating the other, performing multi-objective optimization or conducting different experiments in which different state variables are measured may lead to a unique solution in the second case.

To investigate the non-uniqueness of the inverse problem further, the two-dimensional parameter response surfaces were constructed and analyzed:

#### Two-dimensional parameter response surfaces

The uniqueness of the inverse problem was evaluated by constructing two-dimensional parameter response surfaces of the objective function, Φ(frap¯
 MathType@MTEF@5@5@+=feaafiart1ev1aaatCvAUfKttLearuWrP9MDH5MBPbIqV92AaeXatLxBI9gBaebbnrfifHhDYfgasaacH8akY=wiFfYdH8Gipec8Eeeu0xXdbba9frFj0=OqFfea0dXdd9vqai=hGuQ8kuc9pgc9s8qqaq=dirpe0xb9q8qiLsFr0=vr0=vr0dc8meaabaqaciaacaGaaeqabaqabeGadaaakeaadaqdaaqaaiabdAgaMjabdkhaYjabdggaHjabdchaWbaaaaa@3233@), as a function of pairs of parameters being optimized. The objective function was calculated for three parameter planes: *D*_*f *_- Ka*
 MathType@MTEF@5@5@+=feaafiart1ev1aaatCvAUfKttLearuWrP9MDH5MBPbIqV92AaeXatLxBI9gBaebbnrfifHhDYfgasaacH8akY=wiFfYdH8Gipec8Eeeu0xXdbba9frFj0=OqFfea0dXdd9vqai=hGuQ8kuc9pgc9s8qqaq=dirpe0xb9q8qiLsFr0=vr0=vr0dc8meaabaqaciaacaGaaeqabaqabeGadaaakeaacqWGlbWsdaqhaaWcbaGaemyyaegabaGaeiOkaOcaaaaa@301F@, *D*_*f*_- *K*_*d*_, and Ka*
 MathType@MTEF@5@5@+=feaafiart1ev1aaatCvAUfKttLearuWrP9MDH5MBPbIqV92AaeXatLxBI9gBaebbnrfifHhDYfgasaacH8akY=wiFfYdH8Gipec8Eeeu0xXdbba9frFj0=OqFfea0dXdd9vqai=hGuQ8kuc9pgc9s8qqaq=dirpe0xb9q8qiLsFr0=vr0=vr0dc8meaabaqaciaacaGaaeqabaqabeGadaaakeaacqWGlbWsdaqhaaWcbaGaemyyaegabaGaeiOkaOcaaaaa@301F@ - *K*_*d*_. The response surfaces were calculated using a rectangular grid. The domain of each parameter was discretized into 100 discrete points resulting in 10000 grid points for each response surface plot.

**Figure 4:**
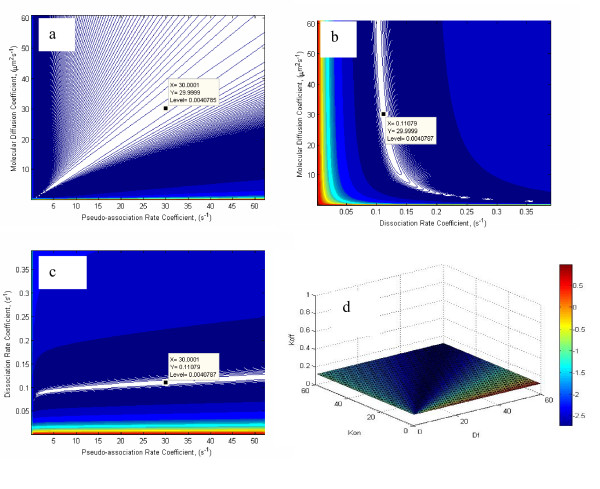
Contours of the objective function, Φ(frap¯
 MathType@MTEF@5@5@+=feaafiart1ev1aaatCvAUfKttLearuWrP9MDH5MBPbIqV92AaeXatLxBI9gBaebbnrfifHhDYfgasaacH8akY=wiFfYdH8Gipec8Eeeu0xXdbba9frFj0=OqFfea0dXdd9vqai=hGuQ8kuc9pgc9s8qqaq=dirpe0xb9q8qiLsFr0=vr0=vr0dc8meaabaqaciaacaGaaeqabaqabeGadaaakeaadaqdaaqaaiabdAgaMjabdkhaYjabdggaHjabdchaWbaaaaa@3233@), in a) *D*_*f *_- Ka*
 MathType@MTEF@5@5@+=feaafiart1ev1aaatCvAUfKttLearuWrP9MDH5MBPbIqV92AaeXatLxBI9gBaebbnrfifHhDYfgasaacH8akY=wiFfYdH8Gipec8Eeeu0xXdbba9frFj0=OqFfea0dXdd9vqai=hGuQ8kuc9pgc9s8qqaq=dirpe0xb9q8qiLsFr0=vr0=vr0dc8meaabaqaciaacaGaaeqabaqabeGadaaakeaacqWGlbWsdaqhaaWcbaGaemyyaegabaGaeiOkaOcaaaaa@301F@, b) *D*_*f *_- *K*_*d*_, c) Ka*
 MathType@MTEF@5@5@+=feaafiart1ev1aaatCvAUfKttLearuWrP9MDH5MBPbIqV92AaeXatLxBI9gBaebbnrfifHhDYfgasaacH8akY=wiFfYdH8Gipec8Eeeu0xXdbba9frFj0=OqFfea0dXdd9vqai=hGuQ8kuc9pgc9s8qqaq=dirpe0xb9q8qiLsFr0=vr0=vr0dc8meaabaqaciaacaGaaeqabaqabeGadaaakeaacqWGlbWsdaqhaaWcbaGaemyyaegabaGaeiOkaOcaaaaa@301F@ - *K*_*d*_, and d) *D*_*f *_- Ka*
 MathType@MTEF@5@5@+=feaafiart1ev1aaatCvAUfKttLearuWrP9MDH5MBPbIqV92AaeXatLxBI9gBaebbnrfifHhDYfgasaacH8akY=wiFfYdH8Gipec8Eeeu0xXdbba9frFj0=OqFfea0dXdd9vqai=hGuQ8kuc9pgc9s8qqaq=dirpe0xb9q8qiLsFr0=vr0=vr0dc8meaabaqaciaacaGaaeqabaqabeGadaaakeaacqWGlbWsdaqhaaWcbaGaemyyaegabaGaeiOkaOcaaaaa@301F@ - *K*_*d *_planes for the synthetic data. The response surfaces were generated using a rectangular grid. The domain of each parameter was discretized into 100 discrete points resulting in 10000 grid points for each response surface plot.

Figures 4a, 4b, 4c, and 4d present response surface plots of the objective function Φ(frap¯
 MathType@MTEF@5@5@+=feaafiart1ev1aaatCvAUfKttLearuWrP9MDH5MBPbIqV92AaeXatLxBI9gBaebbnrfifHhDYfgasaacH8akY=wiFfYdH8Gipec8Eeeu0xXdbba9frFj0=OqFfea0dXdd9vqai=hGuQ8kuc9pgc9s8qqaq=dirpe0xb9q8qiLsFr0=vr0=vr0dc8meaabaqaciaacaGaaeqabaqabeGadaaakeaadaqdaaqaaiabdAgaMjabdkhaYjabdggaHjabdchaWbaaaaa@3233@). The *D*_*f *_- Ka*
 MathType@MTEF@5@5@+=feaafiart1ev1aaatCvAUfKttLearuWrP9MDH5MBPbIqV92AaeXatLxBI9gBaebbnrfifHhDYfgasaacH8akY=wiFfYdH8Gipec8Eeeu0xXdbba9frFj0=OqFfea0dXdd9vqai=hGuQ8kuc9pgc9s8qqaq=dirpe0xb9q8qiLsFr0=vr0=vr0dc8meaabaqaciaacaGaaeqabaqabeGadaaakeaacqWGlbWsdaqhaaWcbaGaemyyaegabaGaeiOkaOcaaaaa@301F@ plane in Figure 4a indicates a well-defined valley, which starts at low values of both parameters and extends linearly to the entire parameter domain. Figure 4a clearly shows a linear relationship between the molecular diffusion coefficient and the pseudo-association rate coefficient, which confirms the high intercorrelation between them, and therefore indicates the difficulty in finding unique values for them. Indeed, an infinite number of combinations of the parameters *D*_*f *_and Ka*
 MathType@MTEF@5@5@+=feaafiart1ev1aaatCvAUfKttLearuWrP9MDH5MBPbIqV92AaeXatLxBI9gBaebbnrfifHhDYfgasaacH8akY=wiFfYdH8Gipec8Eeeu0xXdbba9frFj0=OqFfea0dXdd9vqai=hGuQ8kuc9pgc9s8qqaq=dirpe0xb9q8qiLsFr0=vr0=vr0dc8meaabaqaciaacaGaaeqabaqabeGadaaakeaacqWGlbWsdaqhaaWcbaGaemyyaegabaGaeiOkaOcaaaaa@301F@ (inside the valley) can give almost the same objective function value and produce an excellent fit. This can be confirmed by a slice of three-dimensional parameter hyper-space in the *D*_*f *_- Ka*
 MathType@MTEF@5@5@+=feaafiart1ev1aaatCvAUfKttLearuWrP9MDH5MBPbIqV92AaeXatLxBI9gBaebbnrfifHhDYfgasaacH8akY=wiFfYdH8Gipec8Eeeu0xXdbba9frFj0=OqFfea0dXdd9vqai=hGuQ8kuc9pgc9s8qqaq=dirpe0xb9q8qiLsFr0=vr0=vr0dc8meaabaqaciaacaGaaeqabaqabeGadaaakeaacqWGlbWsdaqhaaWcbaGaemyyaegabaGaeiOkaOcaaaaa@301F@ - *K*_*d *_directions (*K*_*on *_and *K*_*off *_were used for Ka*
 MathType@MTEF@5@5@+=feaafiart1ev1aaatCvAUfKttLearuWrP9MDH5MBPbIqV92AaeXatLxBI9gBaebbnrfifHhDYfgasaacH8akY=wiFfYdH8Gipec8Eeeu0xXdbba9frFj0=OqFfea0dXdd9vqai=hGuQ8kuc9pgc9s8qqaq=dirpe0xb9q8qiLsFr0=vr0=vr0dc8meaabaqaciaacaGaaeqabaqabeGadaaakeaacqWGlbWsdaqhaaWcbaGaemyyaegabaGaeiOkaOcaaaaa@301F@ and *K*_*d *_in the graph, respectively) presented in Figure 4d (the plot is scaled logarithmically). Note that the value of *K*_*d *_is fixed on the known value (0.1108s^-1^). The dark blue area on the slice has the same error level (objective function) indicating that any combination of *D*_*f *_and Ka*
 MathType@MTEF@5@5@+=feaafiart1ev1aaatCvAUfKttLearuWrP9MDH5MBPbIqV92AaeXatLxBI9gBaebbnrfifHhDYfgasaacH8akY=wiFfYdH8Gipec8Eeeu0xXdbba9frFj0=OqFfea0dXdd9vqai=hGuQ8kuc9pgc9s8qqaq=dirpe0xb9q8qiLsFr0=vr0=vr0dc8meaabaqaciaacaGaaeqabaqabeGadaaakeaacqWGlbWsdaqhaaWcbaGaemyyaegabaGaeiOkaOcaaaaa@301F@ on this region will produce the same error and hence the inverse problem is ill-posed. Both Figures show a strong linear positive correlation between *D*_*f *_and Ka*
 MathType@MTEF@5@5@+=feaafiart1ev1aaatCvAUfKttLearuWrP9MDH5MBPbIqV92AaeXatLxBI9gBaebbnrfifHhDYfgasaacH8akY=wiFfYdH8Gipec8Eeeu0xXdbba9frFj0=OqFfea0dXdd9vqai=hGuQ8kuc9pgc9s8qqaq=dirpe0xb9q8qiLsFr0=vr0=vr0dc8meaabaqaciaacaGaaeqabaqabeGadaaakeaacqWGlbWsdaqhaaWcbaGaemyyaegabaGaeiOkaOcaaaaa@301F@ confirming the result of the parameter variance-covariance matrix.

The contours of the objective function in *D*_*f *_- *K*_*d *_and Ka*
 MathType@MTEF@5@5@+=feaafiart1ev1aaatCvAUfKttLearuWrP9MDH5MBPbIqV92AaeXatLxBI9gBaebbnrfifHhDYfgasaacH8akY=wiFfYdH8Gipec8Eeeu0xXdbba9frFj0=OqFfea0dXdd9vqai=hGuQ8kuc9pgc9s8qqaq=dirpe0xb9q8qiLsFr0=vr0=vr0dc8meaabaqaciaacaGaaeqabaqabeGadaaakeaacqWGlbWsdaqhaaWcbaGaemyyaegabaGaeiOkaOcaaaaa@301F@ - *K*_*d *_planes are presented in Figures 4b and 4c. Figure 4b indicates that for small values of the dissociation rate coefficient, the objective function is not sensitive to the molecular diffusion coefficient, which yields an elongated valley in the *D*_*f *_direction. As *K*_*d *_increases the objective function becomes sensitive to the changes in the free molecular diffusion coefficient, which makes it possible to identify this parameter. For large values of *K*_*d*_, the objective function becomes insensitive to the dissociation rate coefficient, which produces an elongated valley in the *K*_*d *_direction. In a small region where the objective function is sensitive to both parameters, it is possible to identify both parameters. Parameter optimization in this zone will produce small estimation variance and narrow confidence intervals.

The contours of the objective function in Ka*
 MathType@MTEF@5@5@+=feaafiart1ev1aaatCvAUfKttLearuWrP9MDH5MBPbIqV92AaeXatLxBI9gBaebbnrfifHhDYfgasaacH8akY=wiFfYdH8Gipec8Eeeu0xXdbba9frFj0=OqFfea0dXdd9vqai=hGuQ8kuc9pgc9s8qqaq=dirpe0xb9q8qiLsFr0=vr0=vr0dc8meaabaqaciaacaGaaeqabaqabeGadaaakeaacqWGlbWsdaqhaaWcbaGaemyyaegabaGaeiOkaOcaaaaa@301F@ - *K*_*d *_plane (Figure 4c) shows that the objective function is not sensitive to the pseudo-association rate coefficient when Ka*
 MathType@MTEF@5@5@+=feaafiart1ev1aaatCvAUfKttLearuWrP9MDH5MBPbIqV92AaeXatLxBI9gBaebbnrfifHhDYfgasaacH8akY=wiFfYdH8Gipec8Eeeu0xXdbba9frFj0=OqFfea0dXdd9vqai=hGuQ8kuc9pgc9s8qqaq=dirpe0xb9q8qiLsFr0=vr0=vr0dc8meaabaqaciaacaGaaeqabaqabeGadaaakeaacqWGlbWsdaqhaaWcbaGaemyyaegabaGaeiOkaOcaaaaa@301F@ increases but becomes more sensitive to this parameter when Ka*
 MathType@MTEF@5@5@+=feaafiart1ev1aaatCvAUfKttLearuWrP9MDH5MBPbIqV92AaeXatLxBI9gBaebbnrfifHhDYfgasaacH8akY=wiFfYdH8Gipec8Eeeu0xXdbba9frFj0=OqFfea0dXdd9vqai=hGuQ8kuc9pgc9s8qqaq=dirpe0xb9q8qiLsFr0=vr0=vr0dc8meaabaqaciaacaGaaeqabaqabeGadaaakeaacqWGlbWsdaqhaaWcbaGaemyyaegabaGaeiOkaOcaaaaa@301F@ decreases. In very low values of the dissociation rate coefficient, the objective function becomes less sensitive to *K*_*d*_. When both parameters are small, there are good chances to identify them with less uncertainty.

**Figure 5:**
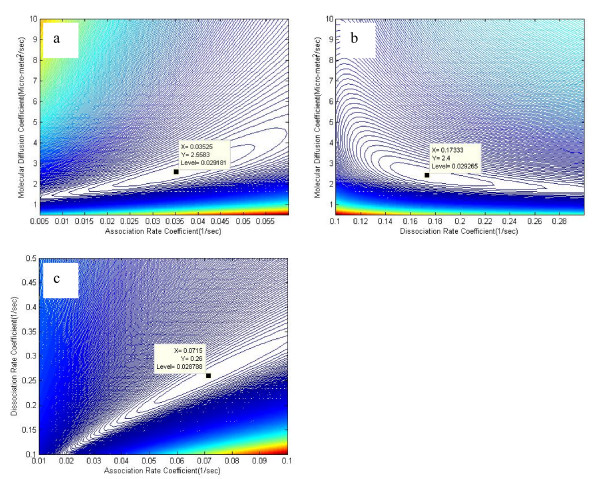
Contours of the objective function, Φ(frap¯
 MathType@MTEF@5@5@+=feaafiart1ev1aaatCvAUfKttLearuWrP9MDH5MBPbIqV92AaeXatLxBI9gBaebbnrfifHhDYfgasaacH8akY=wiFfYdH8Gipec8Eeeu0xXdbba9frFj0=OqFfea0dXdd9vqai=hGuQ8kuc9pgc9s8qqaq=dirpe0xb9q8qiLsFr0=vr0=vr0dc8meaabaqaciaacaGaaeqabaqabeGadaaakeaadaqdaaqaaiabdAgaMjabdkhaYjabdggaHjabdchaWbaaaaa@3233@), in a) *D*_*f *_- Ka*
 MathType@MTEF@5@5@+=feaafiart1ev1aaatCvAUfKttLearuWrP9MDH5MBPbIqV92AaeXatLxBI9gBaebbnrfifHhDYfgasaacH8akY=wiFfYdH8Gipec8Eeeu0xXdbba9frFj0=OqFfea0dXdd9vqai=hGuQ8kuc9pgc9s8qqaq=dirpe0xb9q8qiLsFr0=vr0=vr0dc8meaabaqaciaacaGaaeqabaqabeGadaaakeaacqWGlbWsdaqhaaWcbaGaemyyaegabaGaeiOkaOcaaaaa@301F@, b) *D*_*f *_- *K*_*d*_, and c) Ka*
 MathType@MTEF@5@5@+=feaafiart1ev1aaatCvAUfKttLearuWrP9MDH5MBPbIqV92AaeXatLxBI9gBaebbnrfifHhDYfgasaacH8akY=wiFfYdH8Gipec8Eeeu0xXdbba9frFj0=OqFfea0dXdd9vqai=hGuQ8kuc9pgc9s8qqaq=dirpe0xb9q8qiLsFr0=vr0=vr0dc8meaabaqaciaacaGaaeqabaqabeGadaaakeaacqWGlbWsdaqhaaWcbaGaemyyaegabaGaeiOkaOcaaaaa@301F@ - *K*_*d *_planes for GFP-GR (Scenario A) in lower subspace of model parameters. The response surfaces were generated using a rectangular grid. The domain of each parameter was discretized into 100 discrete points resulting in 10000 grid points for each response surface plot. Intensity scale is the same as Figure 4.

Figure 4a shows several apparent local minima when both the free molecular diffusion coefficient and the pseudo-association rate parameter are small. To investigate the possibility of obtaining a local minimum for inverse problem further when the model parameters are small, one of the possible solutions (*D*_*f *_= 3 *μm*^2^*s*^-1^, Ka*
 MathType@MTEF@5@5@+=feaafiart1ev1aaatCvAUfKttLearuWrP9MDH5MBPbIqV92AaeXatLxBI9gBaebbnrfifHhDYfgasaacH8akY=wiFfYdH8Gipec8Eeeu0xXdbba9frFj0=OqFfea0dXdd9vqai=hGuQ8kuc9pgc9s8qqaq=dirpe0xb9q8qiLsFr0=vr0=vr0dc8meaabaqaciaacaGaaeqabaqabeGadaaakeaacqWGlbWsdaqhaaWcbaGaemyyaegabaGaeiOkaOcaaaaa@301F@ = 0.03*s*^-1^, and *K*_*d *_= 0.1824s^-1^) was used to construct response surfaces. The results are depicted in Figures 5a, 5b, and 5c. As these figures show, there are good possibilities for finding a local minimum in lower subspace of parameters. This is in contrast with the findings of [14], which reported very high values (Ka*
 MathType@MTEF@5@5@+=feaafiart1ev1aaatCvAUfKttLearuWrP9MDH5MBPbIqV92AaeXatLxBI9gBaebbnrfifHhDYfgasaacH8akY=wiFfYdH8Gipec8Eeeu0xXdbba9frFj0=OqFfea0dXdd9vqai=hGuQ8kuc9pgc9s8qqaq=dirpe0xb9q8qiLsFr0=vr0=vr0dc8meaabaqaciaacaGaaeqabaqabeGadaaakeaacqWGlbWsdaqhaaWcbaGaemyyaegabaGaeiOkaOcaaaaa@301F@ = 500s^-1^, *K*_*d *_= 86.4s^-1^) for these parameters (See run 20 in Table 1).

The important findings from the analysis of the two-dimensional parameter response surfaces can be summarized as:

First, response surfaces, though very useful in analyzing the identifiability of the parameters being optimized, are only two-dimensional cross-sections of a full *p – dimensional *parameter hyper-space. The bound response surface does not automatically guarantee a unique solution for the inverse problem. Other local minima or even a global minimum may exist in different regions of the parameter space that do not show up in the response surfaces. Even a well-defined minimum in one part of a two-dimensional plane does not automatically guarantee that no other minima exist and that the inverse problem is unique.

Second, the behavior of the objective function varies between different sub-spaces of the parameter domain. The *D*_*f *_- Ka*
 MathType@MTEF@5@5@+=feaafiart1ev1aaatCvAUfKttLearuWrP9MDH5MBPbIqV92AaeXatLxBI9gBaebbnrfifHhDYfgasaacH8akY=wiFfYdH8Gipec8Eeeu0xXdbba9frFj0=OqFfea0dXdd9vqai=hGuQ8kuc9pgc9s8qqaq=dirpe0xb9q8qiLsFr0=vr0=vr0dc8meaabaqaciaacaGaaeqabaqabeGadaaakeaacqWGlbWsdaqhaaWcbaGaemyyaegabaGaeiOkaOcaaaaa@301F@ and *D*_*f *_- *K*_*d *_planes, for instance, are almost mirror images of each other in the lower space of the parameter domain while in the upper subspace of the parameter domain the *D*_*f *_- Ka*
 MathType@MTEF@5@5@+=feaafiart1ev1aaatCvAUfKttLearuWrP9MDH5MBPbIqV92AaeXatLxBI9gBaebbnrfifHhDYfgasaacH8akY=wiFfYdH8Gipec8Eeeu0xXdbba9frFj0=OqFfea0dXdd9vqai=hGuQ8kuc9pgc9s8qqaq=dirpe0xb9q8qiLsFr0=vr0=vr0dc8meaabaqaciaacaGaaeqabaqabeGadaaakeaacqWGlbWsdaqhaaWcbaGaemyyaegabaGaeiOkaOcaaaaa@301F@ plane shows a strong positive linear relationship.

Third, several small local minima in the two-dimensional plane may be produced by minor oscillations of the numerical simulator. Care should be exercised in interpreting these minima.

## Conclusion

The following results can be drawn from this study:

1. The FRAP protocol provides enough information to estimate one parameter uniquely.

2. Coupling experimental FRAP data with the parameter optimization methodology, one can uniquely estimate the individual values of binding rate coefficients if the molecular diffusion coefficient of biomolecule is known. Given the value of the pseudo-association rate parameter, one can also uniquely identify the molecular diffusion coefficient and dissociation rate parameters simultaneously.

3. The FRAP experiment provides insufficient information for unique simultaneous identification of the molecular diffusion coefficient and pseudo-association rate coefficient. One needs to know one of them and try to estimate the other from the FRAP data using the proposed inverse modeling strategy.

4. One possible approach to estimating the mass transport and binding rate parameters uniquely from the FRAP protocol is to conduct two FRAP experiments on the same class of macromolecule and cell. One experiment may be used to measure the molecular diffusion coefficient of the biomolecule independent of binding in an *effective diffusion *regime. A way to perform this is to use a biomolecule of the same molecular weight and class as the biomolecule under study, which does not react with the vacant binding site(s). Having determined the diffusion coefficient, one can determine the individual values of the reaction rate coefficients in another FRAP experiment conducted in *reaction dominant *or *reaction-diffusion *regimes.

## Appendix

In the present study the inverse problem was treated as a nonlinear optimization problem in which model parameters (*D*_*f*_, Ka*
 MathType@MTEF@5@5@+=feaafiart1ev1aaatCvAUfKttLearuWrP9MDH5MBPbIqV92AaeXatLxBI9gBaebbnrfifHhDYfgasaacH8akY=wiFfYdH8Gipec8Eeeu0xXdbba9frFj0=OqFfea0dXdd9vqai=hGuQ8kuc9pgc9s8qqaq=dirpe0xb9q8qiLsFr0=vr0=vr0dc8meaabaqaciaacaGaaeqabaqabeGadaaakeaacqWGlbWsdaqhaaWcbaGaemyyaegabaGaeiOkaOcaaaaa@301F@, and *K*_*d*_) were estimated by minimizing an appropriate objective function that represents the discrepancy between observed and predicted FRAP. When the measurement errors asymptotically follow a multivariate normal distribution with zero mean and covariance matrix, *V*, the likelihood function, *L*(*β*), can be formulated as [37]:

L(β)=(2π)−N/2det⁡[V]−1/2exp⁡[−12(U*−U(β))TV−1(U*−U(β))]     (A1)
 MathType@MTEF@5@5@+=feaafiart1ev1aaatCvAUfKttLearuWrP9MDH5MBPbIqV92AaeXatLxBI9gBaebbnrfifHhDYfgasaacH8akY=wiFfYdH8Gipec8Eeeu0xXdbba9frFj0=OqFfea0dXdd9vqai=hGuQ8kuc9pgc9s8qqaq=dirpe0xb9q8qiLsFr0=vr0=vr0dc8meaabaqaciaacaGaaeqabaqabeGadaaakeaacqWGmbatdaqadaqaaGGaciab=j7aIbGaayjkaiaawMcaaiabg2da9maabmaabaGaeGOmaiJae8hWdahacaGLOaGaayzkaaWaaWbaaSqabeaacqGHsislcqWGobGtcqGGVaWlcqaIYaGmaaGccyGGKbazcqGGLbqzcqGG0baDdaWadaqaaiabdAfawbGaay5waiaaw2faamaaCaaaleqabaGaeyOeI0IaeGymaeJaei4la8IaeGOmaidaaOGagiyzauMaeiiEaGNaeiiCaaNaei4waSLaeyOeI0YaaSaaaeaacqaIXaqmaeaacqaIYaGmaaGaeiikaGIaemyvau1aaWbaaSqabeaacqGGQaGkaaGccqGHsislcqWGvbqvcqGGOaakcqWFYoGycqGGPaqkcqGGPaqkdaahaaWcbeqaaiabdsfaubaakiabdAfawnaaCaaaleqabaGaeyOeI0IaeGymaedaaOGaeiikaGIaemyvau1aaWbaaSqabeaacqGGQaGkaaGccqGHsislcqWGvbqvcqGGOaakcqWFYoGycqGGPaqkcqGGPaqkcqGGDbqxcaWLjaGaaCzcaiabcIcaOiabbgeabjabigdaXiabcMcaPaaa@6B7E@

where *N *is number of observations, *β *is the vector of parameters being optimized, U* is a vector of observations (e.g. experimental data from FRAP), and *U *is a corresponding vector of model predictions as a function of the parameters being optimized (obtained by solving the forward problem). In this approach the likelihood function is defined as the joint probability density function of the observations and is considered a function of the unknown parameters. The maximum likelihood estimator is the vector of unknown parameters that maximize the magnitude of the same likelihood function [37,38]. Since a logarithm is a monotonic increasing function of its argument, the value of *β *that maximizes *L*(*β*) also maximizes ln *L*(*β*). This basic property of logarithms is often used in optimization studies since ln *L*(*β*) is simpler and much easier to use than *L*(*β*) itself. Therefore equation (6) can be written as:

ln⁡L(β)=−N2ln⁡(2π)−12det⁡[V]−12(U*−U(β))TV−1(U*−U(β))]     (A2)
 MathType@MTEF@5@5@+=feaafiart1ev1aaatCvAUfKttLearuWrP9MDH5MBPbIqV92AaeXatLxBI9gBaebbnrfifHhDYfgasaacH8akY=wiFfYdH8Gipec8Eeeu0xXdbba9frFj0=OqFfea0dXdd9vqai=hGuQ8kuc9pgc9s8qqaq=dirpe0xb9q8qiLsFr0=vr0=vr0dc8meaabaqaciaacaGaaeqabaqabeGadaaakeaacyGGSbaBcqGGUbGBcqWGmbatdaqadaqaaGGaciab=j7aIbGaayjkaiaawMcaaiabg2da9iabgkHiTmaalaaabaGaemOta4eabaGaeGOmaidaaiGbcYgaSjabc6gaUnaabmaabaGaeGOmaiJae8hWdahacaGLOaGaayzkaaGaeyOeI0YaaSaaaeaacqaIXaqmaeaacqaIYaGmaaGagiizaqMaeiyzauMaeiiDaq3aamWaaeaacqWGwbGvaiaawUfacaGLDbaacqGHsisldaWcaaqaaiabigdaXaqaaiabikdaYaaacqGGOaakcqWGvbqvdaahaaWcbeqaaiabcQcaQaaakiabgkHiTiabdwfavjabcIcaOiab=j7aIjabcMcaPiabcMcaPmaaCaaaleqabaGaemivaqfaaOGaemOvay1aaWbaaSqabeaacqGHsislcqaIXaqmaaGccqGGOaakcqWGvbqvdaahaaWcbeqaaiabcQcaQaaakiabgkHiTiabdwfavjabcIcaOiab=j7aIjabcMcaPiabcMcaPiabc2faDjaaxMaacaWLjaGaeiikaGIaeeyqaeKaeGOmaiJaeiykaKcaaa@6980@

In Eqs. (Al) and (A2) the error covariance matrix is defined as:

*V *= *E *[(U* - *U*(*β*))^*T *^(U* - *U*(*β*))]     (A3)

where *E *is the statistical expectation.

The maximum of the likelihood function must satisfy the set of equations:

∂ln⁡L(β)∂β=0     (A4)
 MathType@MTEF@5@5@+=feaafiart1ev1aaatCvAUfKttLearuWrP9MDH5MBPbIqV92AaeXatLxBI9gBaebbnrfifHhDYfgasaacH8akY=wiFfYdH8Gipec8Eeeu0xXdbba9frFj0=OqFfea0dXdd9vqai=hGuQ8kuc9pgc9s8qqaq=dirpe0xb9q8qiLsFr0=vr0=vr0dc8meaabaqaciaacaGaaeqabaqabeGadaaakeaadaWcaaqaaiabgkGi2kGbcYgaSjabc6gaUjabdYeamnaabmaabaacciGae8NSdigacaGLOaGaayzkaaaabaGaeyOaIyRae8NSdigaaiabg2da9iabicdaWiaaxMaacaWLjaGaeiikaGIaeeyqaeKaeGinaqJaeiykaKcaaa@3F25@

When the error covariance matrix is known, maximization of Eq. (A2) is equivalent to the minimization of the following weighted least square problem (i.e. values of *β *that maximize Eq. (A2) also minimize the equation below):

*φ*(*β*) [(U* - *U*(*β*))^*T *^*V*^-1 ^(U* - *U*(*β*))]     (A5)

Furthermore, if information is available about the values and distribution of the parameters being optimized, it can be incorporated in the objective function by modifying it to:

*φ*(*β*) = [(U* - *U*(*β*))^*T*^*V*^-1 ^(U* - *U*(*β*))] + [(β* - β^
 MathType@MTEF@5@5@+=feaafiart1ev1aaatCvAUfKttLearuWrP9MDH5MBPbIqV92AaeXatLxBI9gBaebbnrfifHhDYfgasaacH8akY=wiFfYdH8Gipec8Eeeu0xXdbba9frFj0=OqFfea0dXdd9vqai=hGuQ8kuc9pgc9s8qqaq=dirpe0xb9q8qiLsFr0=vr0=vr0dc8meaabaqaciaacaGaaeqabaqabeGadaaakeaaiiGacuWFYoGygaqcaaaa@2E64@)^*T *^Vβ−1
 MathType@MTEF@5@5@+=feaafiart1ev1aaatCvAUfKttLearuWrP9MDH5MBPbIqV92AaeXatLxBI9gBaebbnrfifHhDYfgasaacH8akY=wiFfYdH8Gipec8Eeeu0xXdbba9frFj0=OqFfea0dXdd9vqai=hGuQ8kuc9pgc9s8qqaq=dirpe0xb9q8qiLsFr0=vr0=vr0dc8meaabaqaciaacaGaaeqabaqabeGadaaakeaacqWGwbGvdaqhaaWcbaacciGae8NSdigabaGaeyOeI0IaeGymaedaaaaa@3193@ (β* - β^
 MathType@MTEF@5@5@+=feaafiart1ev1aaatCvAUfKttLearuWrP9MDH5MBPbIqV92AaeXatLxBI9gBaebbnrfifHhDYfgasaacH8akY=wiFfYdH8Gipec8Eeeu0xXdbba9frFj0=OqFfea0dXdd9vqai=hGuQ8kuc9pgc9s8qqaq=dirpe0xb9q8qiLsFr0=vr0=vr0dc8meaabaqaciaacaGaaeqabaqabeGadaaakeaaiiGacuWFYoGygaqcaaaa@2E64@)]     (A6)

where β* is the parameter vector containing the prior information, β^
 MathType@MTEF@5@5@+=feaafiart1ev1aaatCvAUfKttLearuWrP9MDH5MBPbIqV92AaeXatLxBI9gBaebbnrfifHhDYfgasaacH8akY=wiFfYdH8Gipec8Eeeu0xXdbba9frFj0=OqFfea0dXdd9vqai=hGuQ8kuc9pgc9s8qqaq=dirpe0xb9q8qiLsFr0=vr0=vr0dc8meaabaqaciaacaGaaeqabaqabeGadaaakeaaiiGacuWFYoGygaqcaaaa@2E64@ is the corresponding predicted parameter vector, and *V*_*β *_is the covariance matrix for the parameter vector. This kind of optimization is known as Bayesian estimation. The second term in Eq. (A6), which is sometimes called the plausibility criterion or penalty function, ensures that the optimized values of the parameters remain in some feasible region around β*. Matrices *V*and *V*_*β*_, which are sometimes called weighting matrices, provide information about the measurement accuracy as well as any possible correlation between measurement errors and between parameters [44].

An obvious limitation of Eq. (A6) is that the error covariance matrix is generally not known. A common approach to overcoming this problem is to make some a priori assumptions about the structure of the error covariance matrix. In the absence of any additional information regarding the accuracy of input data, the simplest and most recommended way is to assume that the observation errors are uncorrelated, which implies setting *V *equal to the identity matrix and *V*_*β *_to zero [44]. In this case the optimization problem collapses to the well-known ordinary least squares formulation (Eq. (12)).

Many techniques have been developed in the past to solve nonlinear optimization problems [37,38,44]. These techniques solve Eq. (12) iteratively by first starting with initial guesses at the parameter values and updating them until satisfactory results are obtained. One of the most widely-used optimization algorithms to obtain the search direction is the Levenberg-Marquardt method [45,46]:

Δ*p*^(*k*) ^= -(*J*(*p*^(*k*)^)^*T *^(*J*(*p*^(*k*)^) + *λD*(*p*^(*k*)^)^*T *^*D*(*p*^(*k*)^))^-1 ^*J*(*p*^(*k*)^)^*T*^*r*(*p*^(*k*)^)     (A7)

where *λ *is a positive scalar known as Marquardt's parameter or Lagrange multiplier, *J *is the Jacobian or sensitivity matrix, and *D *is a scaling positive definite matrix that ensures the descent property of the algorithm even if the initial guess is not "smart". For non-zero values of *λ*, the Hessian approximation is always a positive definite matrix, which ensures the descent property of the algorithm.

If *D *is the identity matrix, the Levenberg-Marquardt algorithm interpolates between the steepest descent (*λ *→ +∞) and the Gauss-Newton (*λ *→ 0) methods [38]. The steepest descent scheme is often too inefficient, requiring a large number of iterations that tend to zigzag in a hemstitching pattern, and it is not recommended for optimization [37]. The Gauss-Newton formula, which simply ignores the second term in Eq. (A7) and assumes that *J*(*p*^(*k*)^)^*T*^*J*(*p*^(*k*)^), is a sufficient approximation for the Hessian, and equations (A7), which are the normal equations for Eq. (16), failed to converge to solution in the optimization problem considered in this study. The reason for failure was computation of ill-conditioned *J*(*p*^(*k*)^)^*T*^*J*(*p*^(*k*)^). To avoid this problem, we solved the linear least square problem (Eq. (16)) by *QR *decomposition [36].
